# Reduced Cortical Pyramidal Neuron Membrane Excitability and Synaptic Function in Parkinsonian Mice and Their Restoration by L-Dopa Treatment: Indirect Mediation by Striatal Dopaminergic Activity

**DOI:** 10.3390/brainsci16030285

**Published:** 2026-03-03

**Authors:** Huimin Chen, Manli Zhong, Geng Lin, Francesca-Fang Liao, Fu-Ming Zhou

**Affiliations:** 1 Department of Pharmacology, Addiction Science and Toxicology, College of Medicine, University of Tennessee Health Science Center, 71 South Manassas Street, Memphis, TN 38103, USA; hmchen92@gmail.com (H.C.);; 2 College of Life and Health Sciences, Northeastern University, No. 195, Chuangxin Road, Hunnan District, Shenyang 110169, China; 3 Teaching Center for Basic Medical Experiments, China Medical University, Shenyang 110122, China

**Keywords:** cerebral cortex, cognition, dopamine receptor, inwardly rectifying potassium channel, medium spiny neuron, Parkinson’s disease, pyramidal neuron dendritic spine, striatum

## Abstract

**Background**: We previously established that striatal, but not cortical, dopaminergic activation stimulates movement, indicating that the crucial and original site of dopaminergic stimulation of motor function is the striatum, not the motor cortex. In the present study, we have further investigated the potential effects of the cortical and striatal dopaminergic activity on cortical pyramidal neuron physiology. **Methods and Results**: First, under a constant fluorescence imaging condition, we established that DA innervation and D1R and D2R expression were very low in the cerebral cortex but very high in the striatum. Second, we performed cellular neurophysiological experiments on layer 2/3 pyramidal neurons in the primary motor cortex (M1) in tyrosine hydroxylase gene knockout (TH-KO) DA-depleted mice that have hyperfunctional DA receptors. Using brain slice–whole-cell patch-clamping techniques, we found that M1 layer 2/3 pyramidal neurons had lower input resistance, stronger inward rectification, more negative RMP, and fired fewer spikes in DA-depleted TH-KO mice than in DA-intact WT mice; M1 layer 2/3 pyramidal neurons also had a diminished synaptic release function with reduced frequencies for spontaneous and miniature excitatory synaptic currents in TH-KO mice compared to WT mice. Third, we also found that when TH-KO mice were treated with L-dopa before brain slice preparation, these neurophysiological deficits of M1 layer 2/3 pyramidal neurons were reversed, but 30 min incubation of cortical brain slices with 10–20 μM DA produced no detectable effect in M1 layer 2/3 pyramidal neurons in TH-KO mice and WT mice. Fourth, Golgi staining showed that cortical pyramidal neuron morphology was indistinguishable between WT mice and TH-KO mice. **Conclusions**: Our results indicate that DA loss in the striatum, not in the cortex, indirectly reduces cortical pyramidal neuron membrane excitability and weakens synaptic function. Our data also indicate that (1) the normal direct effects of the cortical DA system on cortical pyramidal neurons are weak, (2) the striatal DA system is the dominant DA system in the brain, and (3) striatal DA activity can indirectly increase cortical neuron activity (spike firing and synaptic activity) and thus critically contribute to brain function. Additionally, our data suggest that in DA depletion rodent PD models, DA loss-induced effects on cortical pyramidal neurons and other neurons are functional rather than structural, such that DA replenishment restores motor function almost instantaneously. These findings provide important insights into how the brain’s dopaminergic system controls our motor and cognitive functions and indicate that the striatum is the main therapeutic target of dopaminergic drugs.

## 1. Introduction

In Parkinson’s disease, degeneration of nigral dopamine (DA) neurons causes a profound loss of brain motor function and thus immense suffering to the patients and eventually early death [1,2,3]. Thus, it is important to understand how DA affects brain neuron activity. Anatomically, DA innervation is intense in the striatum in mammalian animals, including humans, whereas in the cerebral cortex, particularly in rodents, the common experimental animals for studying the brain dopamine system, DA innervation is sparse [4,5,6,7]. Comparative anatomical studies further indicate that rodent motor cortical areas receive particularly low DA innervation [8]. Highly specific and sensitive neurochemical (HPLC) measurement indicates, consistently, that in rodents, the cortical vs. striatal tissue DA content ratio is ~1:70, with the cortical DA level being very low [9,10,11,12]. Similarly, vast differences in cortical vs. striatal DA contents are also convincingly measured in the human brain [13,14]. In parallel to the very low DA level in the cerebral cortex, DA receptor expression is also low in the cerebral cortex but very high in the striatum: the ratio is ~0.1 for cortical D1R and D2R mRNA and D1-like and D2-like receptor levels vs. striatal D1R and D2R mRNA and receptor levels (estimated using the values in references [13,15]; also see reference [16] for autoradiographic data showing the strikingly different levels of D1R and D2R in the striatum and cerebral cortex) [13,15,16]. Our prior functional studies showed that microinjected D1-like or D2-like agonists into the motor cortex did not stimulate motor activity, but microinjection of these same agonists into the striatum stimulated motor activity [17]. Expression of D3Rs and D4Rs (members of the D2-like receptor family) and D5Rs (a member of the D1-like receptor family) is generally low or very low in the brain, with the exception of striatal cholinergic interneurons expressing significant amounts of D5Rs and olfactory tubercle cells expressing significant amounts of D3Rs [18,19]. In the cerebral cortex, D3R, D4R, and D5R mRNA expression levels are lower than those of D1R and D2R mRNAs.

The brain DA system is deeply involved in Parkinson’s disease (loss of motor function is the key symptom) and schizophrenia (dysfunctional cognition is the key symptom) [2,20]. The cerebral cortex is crucial for cognition and motor control, based on data from evolutionary studies and clinicopathological studies of lesion/stroke patients, including patients who received lobotomy [21]. Thus, a critical question has been: How do DA and DA loss each affect membrane excitability and synaptic function of cortical pyramidal neurons? The data collected in the past 40 years from the existing literature are highly contradictory with studies reporting DA and D1R and D2R agonists increasing, decreasing, or having no effect on the membrane excitability and spike firing of same populations of frontal cortical pyramidal neurons in normal animals, including primates (see Supplemental Table S1 for a list of conflicting reports; also see references [22,23]). Reported results from DA lesion studies are also conflicting; for example, one study reported that in MPTP-treated monkeys, pyramidal tract (PT) cortical pyramidal neuron firing rate was decreased, whereas intratelencephalic (IT) corticostriatal pyramidal neuron firing was not altered [24]; in rodents, it was reported that DA depletion decreased IT neuron firing but did not alter PT neuron firing in vivo [25]; however, another study reported that DA depletion reduced PT neuron excitability and did not affect IT neuron excitability examined by brain slice-patch clamp recording [26]. But another patch clamp recording study reported that mouse M1 layer 2/3 and layer 5 pyramidal neuron action potential firing (a few Hz to 20 Hz) evoked by injected currents in the range of 100–300 pA were not altered by 6-OHDA-induced DA depletion [27] (see Supplemental Table S1 for additional conflicting reports). These conflicting results clearly indicate that the long-standing question remains: How does DA affect the physiology of cortical neurons in both normal animals and DA-depleted animals? Our present study seeks to answer this important question. Additionally, we also seek to determine if cortical pyramidal neuron morphology is normal or atrophic in the absence of DA. For this question, the literature currently does not have relevant data from human postmortem PD brains (to our knowledge), and literature data from DA depletion animal PD models are limited and conflicting [28].

## 2. Materials and Methods

### 2.1. Animals

The use of animals was approved by the Institutional Animal Care and Use Committee of the University of Tennessee Health Science Center (UTHSC) in Memphis, Tennessee (protocol #20-0146, approved on 14 May 2020; protocol #23-0438, approved on 15 May 2023). The mice were housed in groups of 5 and had free access to food and water.

**C57BL/6J wild-type mice.** Original breeders of C57BL/6J normal WT mice (stock # 000664) were purchased from the Jackson Laboratory (Bar Harbor, MA, USA). All mice used in this study were on the same C57BL/6J background for more than 20 generations.

**Figure 1:**
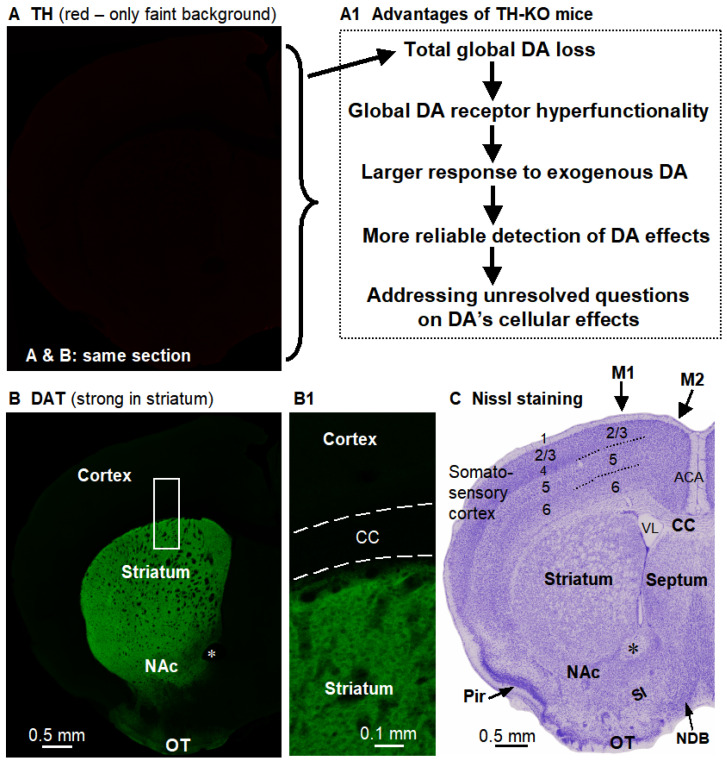
The key brain characteristics of the TH-KO mouse, which is the mouse model for this study. (**A**). DA neurons in TH-KO mice have no TH (no specific red signal, only a very faint non-specific background red signal). (**A1**) lists the crucial advantages of TH-KO mice for studying the DA system. (**B**). DA neurons in these same TH-KO mice remain anatomically intact, as indicated by the apparently normal dopamine transporter (DAT) levels (green signal). Boxed area is displayed in (**B1**). The black holes in the striatum are cortex-originated axonal bundles. (**A**,**B**) are confocal images of a dual-stained same brain section, such that the DAT green signal delineated structures in (**B**) apply to (**A**). (**C**). Nissl-stained section shows the general structures of the brain coronally sectioned at that position; this section was from a different mouse and was slightly posterior to (**B**). ACA, anterior cingulate area/cortex; CC, corpus callosum; M1 and M2, primary and supplementary motor cortices; NAc, nucleus accumbens; NDB, nucleus of diagonal band; OT, olfactory tubercle; Pir, piriform cortex; SI, substantia innominata; VL, lateral ventricle. *, anterior commissure. Nomenclature and demarcation follow the Allen Mouse Brain Reference Atlas.

**Figure 2:**
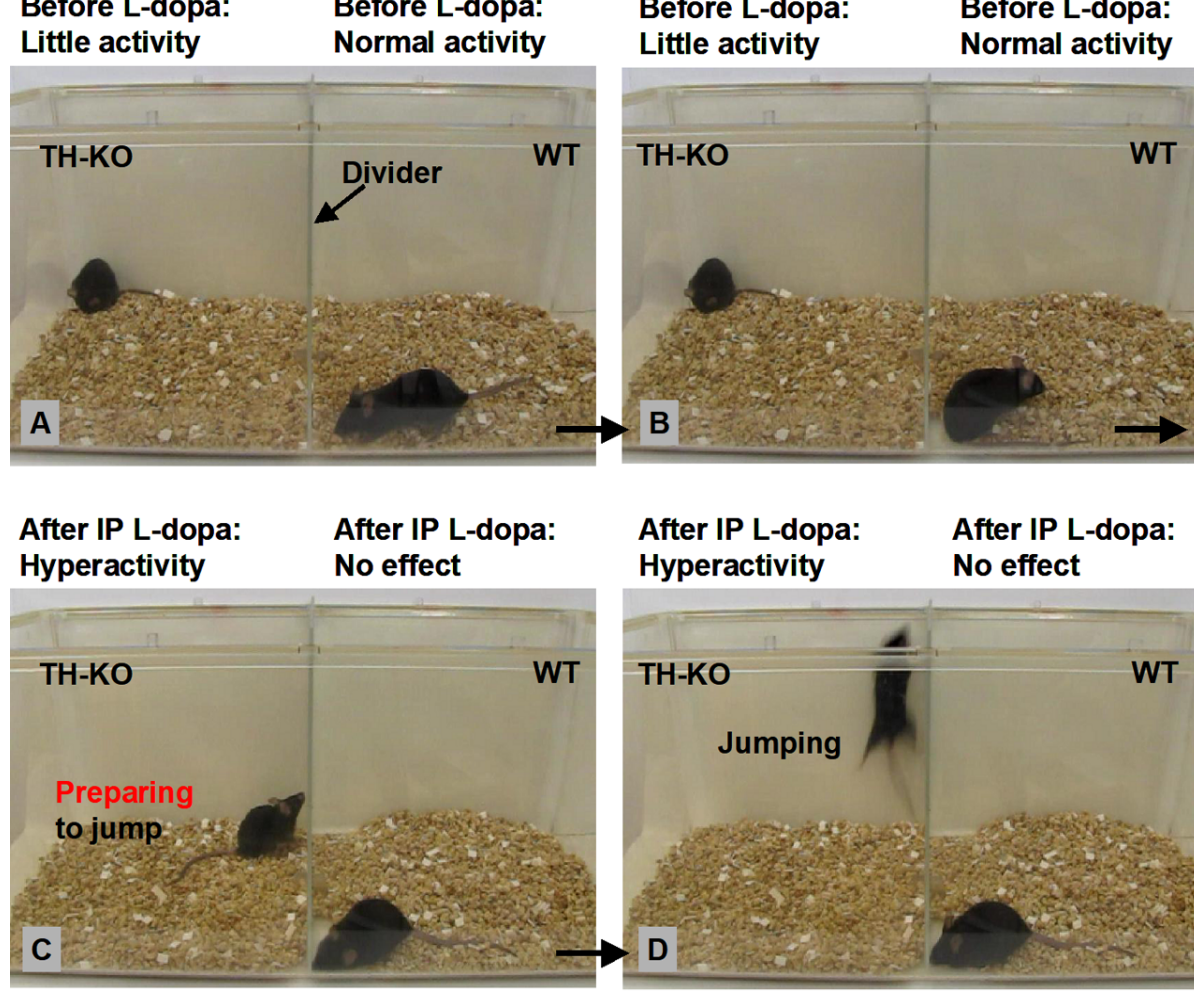
Behavioral characteristics of TH-KO mice and the striking dopaminergic stimulation of movement. (**A**,**B**) show that an example an L-dopa-off TH-KO mouse (male, 7 months of age) who was akinetic and displayed little activity (the daily maintenance doses of 3 mg/kg L-dopa and 3 mg/kg benserazide were injected 24 h previous), while the WT mouse (male, 7 months of age) displayed normal activities (locomotion, digging, chewing, and grooming). In this paper, we use the terms L-dopa-off TH-KO mice and basal TH-KO mice interchangeably. (**C**,**D**) show that ~12 min after IP injection of 10 mg/kg L-dopa (with 5 mg/kg benserazide), the same TH-KO mouse became hyperactive (excessive and fast locomotion, digging, rearing, and jumping—displaying impressive motor capabilities we have never seen in WT mice), whereas the WT mouse displayed no response to the same L-dopa injection. These data demonstrate the ground truth about the profound dopaminergic stimulation of motor function, the hyperfunctional DA receptors in DA-depleted animals (including humans), and the lack of effect of L-dopa treatment in normal animals. Additionally, based on our extensive observations of ~500 TH-KO mice, L-dopa (IP) triggers behavioral activation in every TH-KO mouse, male and female, young (the youngest mice we tested were PN10) and old (the oldest mice we tested were 2.5 years of age); also, 3–12-month-old mice displayed the strongest motoric responses, likely because during this age range (young adult to middle age), the DA system is fully mature (DA receptors have reached the normal maximal expression), neuronal circuits and skeletal systems also have reached their peak function. TH-KO mice of ≥18 months tend to display less vigorous motoric responses because (1) their muscle–skeletal system is declining and aging just like WT mice and humans, and (2) minor needle injuries of long-term daily IP injection of L-dopa (required for TH-KO mice to survive) can accumulate (although we did not perform pathological examination), leading to modest injuries that can cause pain during vigorous physical activities; therefore, these relatively old TH-KO mice run and jump less vigorously to avoid pain. Very young and juvenile TH-KO mice show relatively weak responses because all neuronal and skeletal systems, including the DA receptor expression, have not reached their normal maximal level, just like a young child walking and running slowly and with little ability to jump. The quick restoration of motor and behavioral functions indicates that, except for the lack of DA due to genetic deletion of the TH gene in DA neurons, with daily low-dose L-dopa treatment, these mice (including their CNS and PNS) develop normally. The anatomy of motor control neuronal circuits (including dendritic spines) in L-dopa-off TH-KO mice is also likely normal because of the quick and full restoration of motor function: it is hard to imagine that anatomical deficits can be fully restored quickly (in ~5 min). Therefore, the only pathology in this mouse model is a lack of TH in DA neurons. This is strikingly similar to the accidentally MPTP-poisoned healthy young humans whose motor function was severely impaired by MPTP but profoundly restored by L-dopa treatment [34]. It is also similar to the L-dopa honeymoon—the initial period of highly effective L-dopa treatment when the patient is in early-to-middle stage PD. Late-stage PD may have, in addition to very severe DA neuron loss, other significant pathologies inflicting peripheral, brainstem, and cerebral cortexes, such that L-dopa treatment becomes less effective—motor function is expressed in the context of the good functioning of many brain systems and peripheral systems. Based on our extensive observations, D1 agonist SKF81297 and D2 agonists quinpirole and ropinirole (IP injection) each stimulate motor activity in TH-KO mice. In contrast, in WT mice, IP L-dopa and SKF81297 had no effect, whereas D2 agonists quinpirole and ropinirole each inhibit motor activity likely due to activation of the D2 autoreceptor on DA neurons and hence the inhibition of DA neuron spontaneous firing and DA release. See also our previous study [31] for additional details and interpretations. It is also our experience that while L-dopa can maintain TH-KO mice well, SKF81297 and ropinirole cannot: TH-KO mice deteriorated in their general condition after a few days on SKF81297 or ropinirole.

**Tyrosine hydroxylase (TH) gene KO (TH-/-) mice with total DA loss in all DA neurons of the entire brain**. In these mice, the TH gene in DA neurons is selectively deleted [29,30]. TH, and thus DA, are lacking in the entire brain, including in the striatum and cerebral cortex, but DA neurons and their axons are intact with normal expression of dopamine transporters (Figure 1); consequently, when exogenous L-dopa is provided to bypass the TH step, these DA neurons and axons can make DA from L-dopa. Starting at PN15, TH-KO mice require daily IP injection of 3 mg/kg L-dopa + 3 mg/kg benserazide treatment to survive (benserazide is a dopa decarboxylase inhibitor that does not cross the blood–brain barrier). The lack of DA leads to supersensitive or hyperfunctional D1Rs and D2Rs, as indicated by strong motor activity and c-fos expression in the striatum and other brain areas upon L-dopa treatment [31,32]; an example of the striking behavioral activation is depicted in Figure 2. As demonstrated in our recent study [33], hyperfunctional DA receptors produce larger DA cellular responses that can be more reliably examined.

The original breeders for TH-KO mice were generously donated to Fu-Ming Zhou in 2014 by Dr. Martin Darvas from the University of Washington. TH-KO mice were initially identified using PCR-based genotyping and then were routinely and reliably identified by their baseline akinesia and their robust motor response to IP-injected L-dopa. We have already published 3 research papers using TH-KO mice derived from the same original breeders [31,32,33]. We also need to note here that TH+/− mice are indistinguishable from WT mice in behavior, body size, TH immunostain intensity, and a lack of L-dopa response.

**Figure 3:**
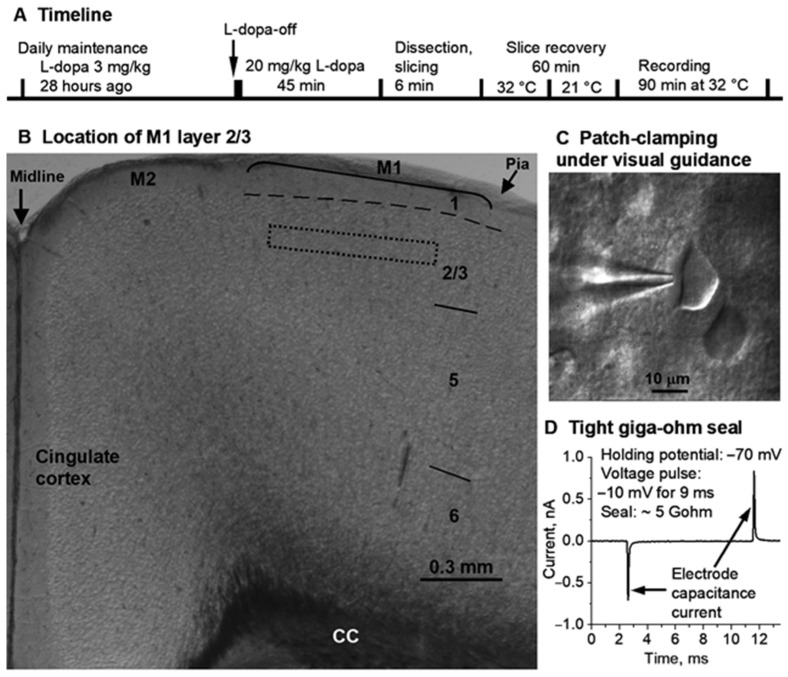
Locating M1 layer 2/3 and patch-clamping M1 layer 2/3 pyramidal neurons. (**A**) is the timeline of L-dopa treatment, brain slice preparation, and recording. 3 mg/kg L-dopa + 3 mg/kg benserazide. 20 mg/kg L-dopa + 5 mg/kg benserazide. IP injection. (**B**) is a mouse frontal cortical slice viewed under a low power (4×) objective with M1 layer 2/3 being identified and located, approximately, because cortical area and layer borderlines (except layer 1) are approximate with considerable overlap and uncertainty, especially in rodents whose cerebral cortex is flat with few landmarks, and the division into areas and layers is for the convenience of description. To maximize the homogeneity of recorded neurons, recording was further restricted to the approximate middle portion of layer 2/3, as indicated by the dashed box. (**C**) is a snapshot of patch-clamping a cortical pyramidal neuron under the guidance of a 60× NA 0.9 water-immersion objective. (**D**) shows the tight giga-ohm (5 GΩ: 2 pA divided by 10 mV) seal formed between the patch electrode tip and the cell membrane immediately before breaking into whole-cell recording mode. The electrode capacitance currents were induced by the 10 mV voltage jump (from the holding potential −70 mV to −80 mV for 9 ms and then returning to the holding potential −70 mV). CC, corpus callosum. M1, primary motor cortex. M2, secondary motor cortex.

**L-dopa treatment (Supplemental Videos S1 and S2):** Upon 20 mg/kg L-dopa + 5 mg/kg benserazide IP injection, motor stimulation and hyperactivity appeared within 10 min, peaked at 30–40 min, plateaued for ~2–3 h, and slowly declined, but moderate motor hyperactivity remained for 6–8 h. These robust behavioral effects have been documented by the creator of TH-KO mice [29,30] and by our own published work [31]. The reader can also easily see the L-dopa-induced robust motor activation in TH-KO mice in Figure 2 and in the Supplemental Videos (quantitative analysis was published in references [29,30,31]). In our present cellular neurophysiology study, we euthanized mice at 45 min after L-dopa injection, dissected the brain quickly (within 1 min and with ice-cold cutting solution poured onto the brain during the process), cut brain slices (5 min), allowed 60 min for the brain slices to recover, and then made patch-clamp recordings of neurons for 90 min, as seen in the timeline in Figure 3A.

**D2-GFP mice.** Heterozygous BAC D2-GFP+/− breeder mice [35] were purchased from the Mutant Mouse Research Resource Centers (MMRRC) ([stock Tg (Drd2-EGFP) S118Gsat/Mmnc (000230-UNC); RRID: MMRRC_000230-UNC]) in 2009. The D2-GFP labeling is robust and highly selective, as reported and confirmed by the creator [35], other scientists [36], and our own work [33,37,38]. D2-GFP mice were crossed to TH-KO mice to first produce D2-GFP-TH+/− mice and eventually TH-KO-D2-GFP mice to accurately identify D2-MSNs in brain slices from TH-KO mice. Mice had free access to food and water. Experimental use of D2-GFP mice and TH-KO-D2-GFP mice was approved by the Institutional Animal Care and Use Committee of the University of Tennessee Health Science Center, Memphis, TN, USA (protocol #20-0146, approved on 14 May 2020; protocol #23-0438, approved on 15 May 2023).

We need to note here that brain and neuronal development are probably normal in TH-KO mice, based on (1) our extensive use and visual observation of TH-KO mice and their brains and (2) published data and conclusions from Richard Palmiter’s lab (the creator of TH-KO mice) [29] and our own published comparative data and conclusions [32,39]. In other words, the behavioral and neurophysiological deficits and L-dopa-induced effects are simple direct dopaminergic effects, not consequences of abnormal brain and neuronal development.

### 2.2. Brain Slice-Patch Clamp Electrophysiology

**Brain slice preparation.** Postnatal day 24–25 male mice were used for two reasons: neurons in brain slices from young animals are better visualized due to low myelination and are more viable, leading to the best quality data; and patch-clamping neurons from mature animals is more difficult and time-consuming. But we fully believe that the dopaminergic effects reported in this paper are qualitatively identical and mediated by identical mechanisms in young and mature animals; the dopaminergic cellular effects are likely larger in mature animals because (1) the dopaminergic behavioral effects are clearly larger in adult TH-KO mice than in PN24-25 TH-KO mice (compare Figure 2 of 7-month-old mice with Supplementary Videos of PN24 mice; see also [30,31], unpublished observations of Fu-Ming Zhou), and (2) in striatal D1-medium spiny neurons, the dopaminergic increase in intrinsic excitability is larger in PN30 mice than in PN20 mice [33]. Inclusion of only male mice is discussed in Section 4.4.

Coronal brain slices containing the primary motor cortex (M1; Figure 2) were prepared for electrophysiological recording. Mice were killed by decapitation, and brains were dissected out quickly and immediately immersed for 2 min in an oxygenated ice-cold cutting solution containing (in mM): 220 glycerol, 2.5 KCl, 1.25 NaH_2_PO_4_, 25 NaHCO_3_, 0.5 CaCl_2_, 7 MgCl_2_, 20 D-glucose, and 0.4 vitamin C. The 300 μm-thick coronal brain slices were cut using a Leica Zero Z VT1200S vibratome (Leica Microsystems, Wetzlar, Germany). The brain slices were transferred to a holding chamber filled with an artificial cerebrospinal fluid (aCSF) containing (in mM): 125 NaCl, 2.5 KCl, 25 NaHCO_3_, 1.25 NaH_2_PO_4_, 2.5 CaCl_2_, 1.3 MgCl_2_, 10 D-glucose, and 0.4 vitamin C. The aCSF was continuously bubbled with 95% O_2_ and 5% CO_2_ to supply oxygen and maintain pH at 7.4. The holding chamber was first maintained at 34 °C for 30 min and then kept at room temperature (21 °C). For recording, a single brain slice was transferred to the recording chamber maintained at 32 °C by a TC324B in-line heating temperature controller (Warner Instruments, Holliston, MA 01746, USA). The recording chamber/brain slice was perfused with the aCSF at 2 mL/min driven by a peristaltic pump.

**Electrophysiology.** This study focused on M1 layer II/III pyramidal neurons that can be identified by their location and shape (Figure 3B,C); their action potentials were typical of pyramidal neurons. Conventional visualized brain slice–whole-cell patch-clamping was performed using an Olympus BX51 WI microscope equipped with Nomarski optics, a 60× NA 0.9 water-immersion objective (Olympus Corporation, Tokyo, Japan), and a Zeiss Axiocam MRm digital camera (Carl Zeiss, Jena, Germany) [33,37,40]. A Multiclamp 700B amplifier, pClamp 9.2 software (Clampex for data acquisition and Clampfit for analysis), and Digidata 1322A interface (Molecular Devices, San Jose, CA, USA) were used to acquire electrophysiological data [33,37,40]. Patch pipettes were pulled from borosilicate glass capillary tubing purchased from Sutter Instruments (Novato, CA, USA, #B150-110-10, OD 1.5 mm, and ID 1.1 mm) using a PC-10 puller (Narishige, Tokyo, Japan), and they had resistances of 2–3 MΩ when filled with a pipette solution (see below).

Two types of intracellular or pipette solutions were used. For recording membrane potential and spiking activity in current-clamp recording mode, we used a KCl-based intracellular solution containing the following (in mM): 135 KCl, 0.5 EGTA, 10 HEPES, 2 Mg-ATP, 0.2 Na-GTP, and 4 Na_2_-phosphocreatine (pH 7.3, 285 mOsm). For recording spontaneous and miniature excitatory postsynaptic currents (sEPSCs and mEPSCs) in voltage-clamp mode, we used a CsCl-based intracellular solution containing (in mM): 67.5 KCl, 67.5 CsCl, 0.5 EGTA, 10 HEPES, 2 Mg-ATP, 0.2 Na-GTP, and 4 Na_2_-phosphocreatine (pH 7.3, 285 mOsm). CsCl inhibits K channels and thus increases the input resistance of the neuron and improves the recording of small current signals (noise current is inversely related to the input resistance). Equally important, we have determined that 67.5 mM CsCl-containing pipet solution allows us to distinguish the different action potentials of regular-firing neurons, pyramidal neurons, and fast-firing GABAergic interneurons within the first 2 min after entering whole-cell mode [38], providing a verification of visually identified pyramidal neurons. Signals were filtered at 10 kHz using the built-in 4-pole low-pass Bessel filter in the patch clamp amplifier and digitized at 20 KHz. All recordings were made at 32 °C, maintained by the automatic temperature controller.

Accurate measurement of the rheobase current (the threshold current to evoke a single action potential) requires small current steps (e.g., 5 pA/step or 1 pA/step) or a current ramp. But these requirements are not absolute because the current pulse duration and ramp speed can affect measured values. We obtained approximate rheobase currents based on data of coarse 30 pA/step current injection, but the comparison of approximate rheobase currents is valid when the measurements are performed uniformly in all experimental groups, and the difference is larger than 30 pA and consistent with differences in RMP and input resistance—our data meet these requirements.

### 2.3. Immunofluorescence Staining and Confocal Imaging

Conventional immunofluorescence methods were used to detect DA axons in the striatum and cerebral cortex [7,32,33,37,41,42,43]. Following 10 min intracardiac perfusion with PBS under overdose ketamine and xylazine (120 mg/kg and 12 mg/kg, IP, respectively), the brains were dissected out and fixed in 4% paraformaldehyde dissolved in phosphate-buffered saline (PBS) at 4 °C overnight (~16 h) and then sectioned (50 μm in thickness) on a vibratome. The free-floating sections were incubated with 1% normal donkey serum and 1% normal bovine serum (cat. # 017-000-121, 001-000-121, Jackson ImmunoResearch, West Groves, PA, USA), and 0.4% Triton X-100 in PBS for 1.5 h at room temperature to block non-specific binding and permeabilize the cell membrane, respectively (all primary and secondary antibodies were diluted in this same blocking and permeabilizing solution). Then, these free-floating sections were incubated for 24 h at room temperature with the primary antibody, a polyclonal tyrosine hydroxylase antibody produced in rabbit (diluted at 1:1000; Novus Biologicals, Littleton, CO, USA; cat. # NB300-109), and then rinsed thoroughly (10 min × 4) in PBS, followed by incubating with a donkey anti-rabbit secondary antibody conjugated with the green Alexa Fluor 488 (diluted at 1:400; Invitrogen, Waltham, MA, USA) for 3 h at room temperature followed by thorough rinsing in PBS (10 min × 4). For D1R staining, the primary D1R antibody is a rat monoclonal antibody purchased from Sigma (cat. # D-187, Sigma, St. Louis, MO, USA; used at 1:500 dilution). Donkey anti-rabbit secondary antibody-green Alexa Fluor 488 and donkey anti-rat secondary antibody-green Alexa Fluor 488 (or red Alexa Fluor 568) were purchased from Invitrogen (cat. # A21206 and cat. # A21208, Invitrogen/Thermo Fisher Scientific, Waltham, MA 02451, USA). To detect DA axons in TH-KO mice, a monoclonal dopamine transporter (DAT) antibody raised in rats (Cat. # MAB369, Millipore Corporation, Billerica, MA, USA) was used at 1:2000 dilution, in combination with the donkey anti-rat secondary antibody conjugated with green Alexa Fluor 488 (cat. # A21206 and cat. # A21208, Invitrogen/Thermo Fisher Scientific, Waltham, MA 02451, USA). The stained brain sections were placed on glass slides, lightly air-dried, cover-slipped, and sealed with nail oil. Confocal microscopic images were acquired using a Zeiss 710 laser scanning confocal microscope with a 20X/NA 0.8 air objective and the associated image acquisition and processing software Zen (Carl Zeiss, Oberkochen, Germany) at the Neuroscience Institute Imaging Center of the University of Tennessee Health Science Center, Memphis, TN, USA.

### 2.4. Golgi Staining

We used the FD Rapid GolgiStain Kit (cat. # PK401, FD Neurotechnologies, Inc., Ellicott City, MD 21041, USA). Following the kit’s detailed protocol, all containers were cleaned and rinsed with purified deionized 18 MΩ water. The impregnation solution was prepared by mixing equal volumes of Solutions A and B 30 h prior to use. Mice were deeply anesthetized with an overdose of ketamine and xylazine (120 and 12 mg/kg, respectively, IP) and decapitated without perfusion (following the manufacturer’s instructions for the stain kit). Brains were removed from the skull quickly and carefully. After rinsing with the purified 18 MΩ water to remove blood from the brain surface, brains were immersed in the impregnation solution in a light-shielded container (impregnation solution was replaced with a fresh one after the first 6 h of immersion), and the container-brain was gently shaken twice a day during the 12-day immersion period. Afterwards, the brains were put in Solution C, which was replaced after 24 h and kept in the dark for an additional 48 h. A VT1200S vibratome (Leica, Germany) was used to cut 120 μm-thick brain tissue sections. These brain tissue sections were mounted onto gelatin-coated microscope slides using Solution C, air-dried in the dark for 36 h, and then stained with the staining solution (Solution D/E, prepared with the following volume ratio: Solution D:Solution E:purified water = 1:1:2). Finally, the mounted and stained brain tissue sections were dehydrated with ethanol, cleared with xylene, and cover-slipped with Permount; the mouse ID of these sections was also concealed. These sections were examined and photographed using a Keyence BZ-X800E microscope-imaging system with 20X/NA 0.8 air objective and 60X/NA 1.4 oil immersion objective (Keyence Corporation of America, Itasca, IL 60143, USA). This imaging system is equipped with a motorized stage and capable of Z serial imaging and capturing parts of neurons located in different focal planes in the 120 μm-thick tissue sections.

### 2.5. Drugs and Other Chemicals

Water-soluble L-3,4-Dihydroxyphenylalanine (L-3-Hydroxytyrosine) methyl ester hydrochloride (Methyl L-DOPA hydrochloride: Sigma cat. # d1507), water-soluble benserazide-HCl (Sigma cat. # B7283), water-soluble dopamine-HCl (Sigma cat. # H8502), and all other drugs and chemicals were purchased from Sigma-Aldrich (St. Louis, MO, USA). Water-soluble L-dopa and benserazide were required for long-term daily intraperitoneal (IP) injection with 0.3 mL syringes fitted with 31-gauge short (6 mm) needles that minimize injuries—the minimal injuries were indicated by the fact that our TH-KO mice with daily L-dopa injection were able to live to 30 months (the longest we tested). Saline IP injection induced no behavioral effect and no in vivo spike-firing change in either WT mice or TH-KO mice (separate data for the Zhou lab; manuscript under preparation). As a standard practice in our lab, in addition to using vitamin C in the aCSF to partially protect dopamine from oxidation by the bubbling 95% O_2_/5% CO_2_, the dopamine-containing aCSF was placed on ice to retard oxidation, and the dopamine-containing aCSF was replaced every 2 h. For recording spontaneous and miniature glutamatergic EPSCs, picrotoxin (stock solution: 200 mM in DMSO; final working concentration in aCSF: 100 μM) was used to block GABA_A_ receptors.

### 2.6. Data Analysis and Statistics

Electrophysiological data were analyzed using Clampfit 9 (Molecular Devices, San Jose, CA, USA). All data were presented as mean ± SEM. Spontaneous synaptic current data were analyzed using Mini Analysis software 6.0 (Synaptosoft, Fort Lee, NJ, USA). Two-way ANOVA or one-way ANOVA was used to compare the changes among different groups, followed by Tukey’s post hoc comparisons test. The Kolmogorov–Smirnov (K-S) test was used to compare cumulative distributions of sEPSCs and mEPSCs. These statistical calculations were performed using Clampfit 9 (Molecular Devices, San Jose, CA, USA) and StatMost 3.6 (DataMost Corp., Los Angeles, CA, USA), and GraphPad Prism 8.4 (GraphPad Software, San Diego, CA, USA).

## 3. Results

### 3.1. Visual Comparison of DA Innervation and D1R and D2R Expression in the Cerebral Cortex and Striatum

**Figure 4:**
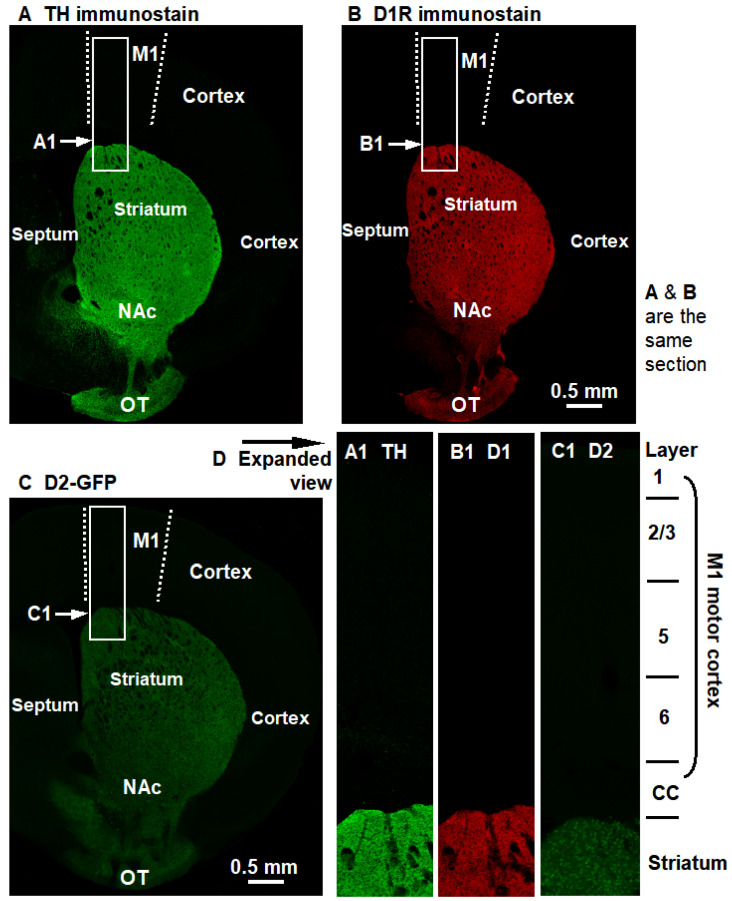
DA innervation and D1R and D2R expression are very high in the striatum but very low in the entire cerebral cortex, including the primary motor cortex (M1). These are important anatomical and molecular facts, but they are usually not presented in the same Figure, and hence the vast differences are not readily appreciated. Here, the three whole-hemisphere pictures are placed side-by-side such that the reader can easily compare the distribution pattern of DA innervation and D1R and D2R expression. (**A**). TH immunostain shows the strikingly intense DA innervation in the striatum and the very low DA innervation in the cerebral cortex. (**B**). D1R immunostain shows the high D1R expression in the striatum and the very low D1R expression in the cerebral cortex. (**C**). D2-GFP, a proxy of D2R, shows the high D2R expression in the striatum and the very low D2R expression in the cerebral cortex. Quantitative values are presented in Table 1. Additionally, D1R and D2R expression signals in TH-KO mice are similar to D1R and D2R signals in WT mice shown here, i.e., D1R and D2R expression remains unchanged but these receptors become hyperfunctional (indicated by L-dopa-triggered supranormal motor function—see Figure 2—and cFos expression—see Figure 1 of our prior publication [32]), via higher binding affinity and/enhanced downstream signaling mechanisms (not fully understood; discussed in reference [44]).

Data from the literature on DA innervation and D1R and D2R expression in the cerebral cortex and striatum were often published in separate papers, and hence, were not convenient for the reader to evaluate, integrate, and appreciate the information. Thus, imaging data on these important anatomical parameters collected from the same animals and displayed side-by-side would be useful and an important contribution to the scientific literature. Additionally, past studies focusing on cortical DA effects imaged cortical DA innervation and DA receptor markers under imaging conditions that oversaturate the same signals in the striatum, leading to confounded and even incorrect data and conclusions. Further, D1-Cre and D2-Cre mice-based viral labeling may produce non-specific positive labeling. For these reasons, and also to base our neurophysiological studies on a solid anatomical foundation, we first broadly characterize DA innervation and D1R and D2R expression in the cerebral cortex and striatum. As shown in Figure 4, we consistently found that DA innervation and D1R and D2R expression are very low in the cerebral cortex but very high in the striatum. These visually striking differences are further quantified and listed in Table 1. These anatomical data perfectly match our published behavioral data: Microinjection of D1R and D2R agonists into the striatum but not the motor cortex induced motor activity in DA-deficient Pitx3 mice [17].

### 3.2. Motor Cortical Pyramidal Neuron Intrinsic Membrane Excitability in L-Dopa-Off TH-KO Mice

**Figure 5:**
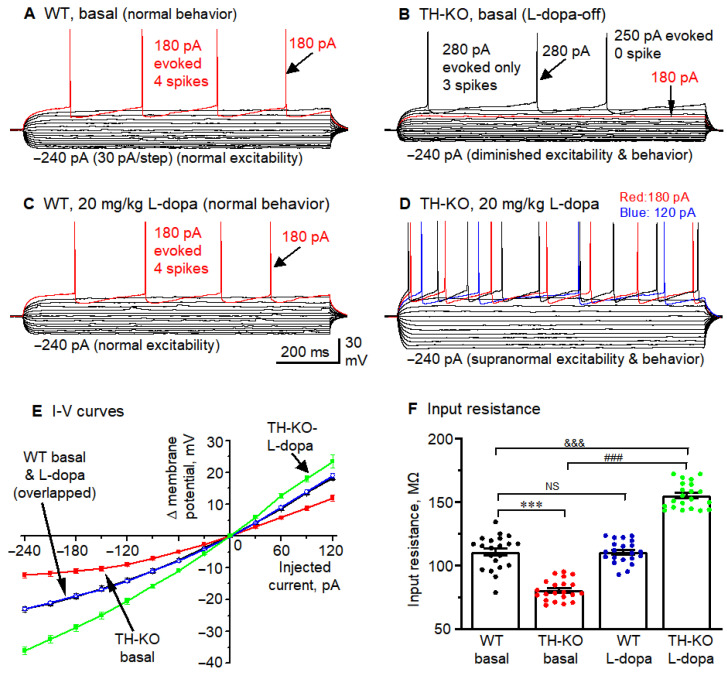
M1 layer 2/3 pyramidal neurons in L-dopa-off TH-KO mice had reduced intrinsic membrane excitability that was reversed by in vivo IP L-dopa treatment. (**A**–**D**): Membrane potential responses of example M1 layer 2/3 pyramidal neurons receiving hyperpolarizing and depolarizing current injection in WT-basal (**A**), TH-KO-basal (L-dopa-off) (**B**), WT-L-dopa (**C**), and TH-KO-L-dopa (**D**). The current pulse started at −240 pA, and the step size was 30 pA for all neurons. Note the lack of difference between (**A**,**C**) and the clear difference between (**B**,**D**), and also the clear differences among (**A**,**B**,**D**). (**E**) Current pulse-induced membrane potential changes in the four groups of mice. (**F**) Whole-cell input resistance of layer 2/3 pyramidal neurons in the four groups of mice. All data showed as mean ± SEM, ***, *p* = 0.0003: WT vs. L-dopa-off TH-KO; ###, *p* = 0.0001: L-dopa-off TH-KO vs. TH-KO-L-dopa; &&&, *p* = 0.0004: WT vs. TH-KO-L-dopa; NS, not significant: WT-basal vs. WT-L-dopa; two-way ANOVA.

We first examined the intrinsic excitability and spike firing of M1 pyramidal neurons in WT mice and L-dopa-off TH-KO mice (Figure 5A,B). The resting membrane potential (RMP), measured in whole-cell mode, was −75.45 ± 0.12 mV (range: −74.7 to −76.8 mV) for WT and −76.83 ± 0.13 mV (range: −75.6 to −78.4 mV) for L-dopa-off TH-KO mice (Table 2). This is a modest (1.4 mV) but statistically significant hyperpolarization for pyramidal neurons in L-dopa-off TH-KO mice, and more importantly, it is physiologically meaningful because a more negative RMP keeps the neurons from reaching the action potential threshold; this effect is compounded with the lower input resistance, substantially reducing spike firing (described in the next paragraph).

Second, we injected a series of current pulses into pyramidal neurons to induce membrane potential changes for measuring whole-cell input resistance (R_In_) and evoking action potential firing (Figure 5A,B). The amplitude of the current pulses started at −240 pA and increased by +30 pA per step, whereas the duration of the current pulses was fixed at 1000 ms. Input resistance was calculated by using Clampfit’s input resistance-calculating function and verified manually by measuring the −30 pA-induced hyperpolarization from the RMP (clustered around −75 mV) and then using Ohm’s law (R_in_ = membrane potential change divided by 30 pA; taking the units into account for the final values). The same −30 pA-induced hyperpolarization was also used to measure the neuronal membrane charging time constant (τ), using the exponential fitting function of Clampfit. τ is directly related to whole-cell input resistance (R_In_) because τ = R_In_ × cell capacitance (cell capacitance is determined by cell membrane surface area, i.e., cell size). Under these conditions, we found that compared to WT mice, layer 2/3 pyramidal neurons of TH-KO-L-dopa-off mice had clearly lower R_In_ and thus required larger depolarizing current injection to trigger action potentials (Figure 5A,B,E,F; Table 2). R_In_ and RMP are the two most important parameters of neuronal intrinsic excitability. Smaller R_In_ and more negative RMP render the neurons less excitable, which was clearly evident in the fewer spikes triggered by the same depolarizing current pulses. In the two example neurons in Figure 5A,B, 180 pA current triggered four action potentials or spikes in the layer 2/3 pyramidal neuron in a WT mouse, whereas the same 180 pA current pulse triggered no spike in the layer 2/3 pyramidal neuron in a L-dopa-off TH-KO mouse. The group differences for spike firing are summarized in Table 2, using these two parameters: the rheobase current and spikes evoked by a 180 pA current pulse—the differences are very clear.

Hyperpolarizing current pulses also revealed inward rectification in M1 cortical pyramidal neurons, i.e., the membrane hyperpolarization became less relative to the amplitude of the injected current, as is clearly seen in the raw membrane potential sweeps (Figure 5A) and in the plots of the injected current pulses vs. the membrane potential changes (Δ mV) (Figure 5E). This is consistent with the inward rectifier K channel Kir2-mediated current being active in cortical pyramidal neurons [45,46,47], similar to the Kir2-mediated membrane potential inward rectification in striatal medium spiny neurons [33,48]. Furthermore, this inward rectification was substantially stronger in L-dopa-off TH-KO mice: hyperpolarizing current pulses induced much smaller hyperpolarization in L-dopa-off TH-KO mice than in WT mice (Figure 5A,B,E), likely because the larger Kir2 current in L-dopa-off TH-KO mice opposes and shunts the hyperpolarizing current pulses and also reduces input resistance and hence membrane charging time, i.e., a broad or global reduction in neuronal intrinsic excitability.

### 3.3. Effects of L-Dopa Treatment on Motor Cortical Pyramidal Neuron Intrinsic Membrane Excitability in TH-KO Mice

Motor cortical pyramidal neurons are a key component of the brain motor control system; brain motor function is also dependent on the DA system, as evidenced by the severely diminished motor function of PD patients and TH-KO mice (Figure 2A,B) and the profound restoration of motor function by L-dopa treatment, even excessive motor activity when L-dopa dose is high, as occurred often in patients during the initial introduction of L-dopa in 1960s [49,50], and it was readily (100% reliability) induced in every TH-KO mouse (Figure 2C,D) and other DA-depleted animals, e.g., in DA-deficient Pitx3 [31,33]. The underlying mechanism for the striking dopaminergic behavioral stimulation in PD patients and DA-depleted animals is that DA receptors very quickly become hyperfunctional, within ~24 h [51].

Here, we tested the potential effects of L-dopa IP treatment on M1 pyramidal neuron excitability in TH KO mice. While TH-KO-L-dopa-off mice were akinetic, these mice started to regain their motor function ~5–6 min after receiving 20 mg/kg L-dopa (IP) and became hyperkinetic at ~10 min, (see also our Supplemental Videos S1 and S2). WT mice show no motor response to L-dopa injection, likely because (1) WT mice have plenty of endogenous L-dopa and L-dopa injection does not increase axon terminal DA level, and (2) DA receptors are normal in WT mice but hyperfunctional in TH-KO mice [30]. We prepared brain slices following the timeline diagrammed in Figure 3A.

Under these experimental conditions, IP injection of 20 mg/kg L-dopa produced no detectable effect on RMP, input resistance, membrane charging τ, or inward rectification and evoked spike firing in M1 layer 2/3 pyramidal neurons of WT mice, just like the lack of any detectable behavioral effect of 20 mg/kg L-dopa IP injection in these mice (Table 2; Figure 5; also see Supplemental Videos S1 and S2; we have published similar behavioral observations [17,31,33]). In contrast, in TH-KO mice, 20 mg/kg L-dopa induced robust motor stimulation, as seen in Supplemental Videos S1 and S2 (see our prior work [31], and also the work of Richard Palmiter’s lab [29,30]). Further, in TH-KO mice, we found that the 20 mg/kg L-dopa treatment increased M1 layer 2/3 pyramidal neuron intrinsic excitability when examined in brain slices prepared from these L-dopa-treated TH-KO mice: RMP was modestly but significantly depolarized to −73.87 ± 0.22 mV, input resistance was substantially increased to 155.1 ± 2.2 MΩ together with membrane charging time constant τ, and evoked spike firing was also substantially increased with a corresponding decrease in rheobase currents for evoking action potentials (Table 2; Figure 5B,D,F). Furthermore, upon 20 mg/kg L-dopa treatment, M1 layer 2/3 pyramidal neurons in TH-KO mice also displayed less inward rectification of membrane potential responses to hyperpolarizing current pulse injections, as shown in the four example neurons (Figure 5A–D); the quantified group I-V curves are shown in Figure 5E,F, and also in Table 2. The strong inward rectification of pyramidal neurons in TH-KO mice was greatly reduced upon L-dopa treatment, whereas the moderate inward rectification of pyramidal neurons in WT mice was not affected by L-dopa treatment. Also, the inward rectification detected here and shown in Figure 5 is identical to the published Kir2-mediated inward rectification in cortical pyramidal neurons [45] and striatal medium spiny neurons [33,48] (more discussion on Kir2-mediated inward rectification and its inhibition by systemic L-dopa treatment in the Discussion Section).

### 3.4. Cortical Glutamatergic Synaptic Transmission in L-Dopa-Off TH-KO Mice and the Effects of L-Dopa Treatment

The data presented above indicate that in L-dopa-off TH-KO mice, M1 pyramidal neurons had lower intrinsic excitability, and this deficiency was restored by in vivo L-dopa treatment. Here, we further examined the excitatory glutamatergic synaptic transmission among M1 pyramidal neurons in brain slices from L-dopa-off TH-KO mice and IP L-dopa-treated TH-KO mice. All recordings were made at a holding potential of −70 mV and in the presence of 100 μM picrotoxin to block GABA_A_ receptor-mediated synaptic currents; for recording mEPSCs, 1 μM TTX was also present to block action potentials.

**Figure 6:**
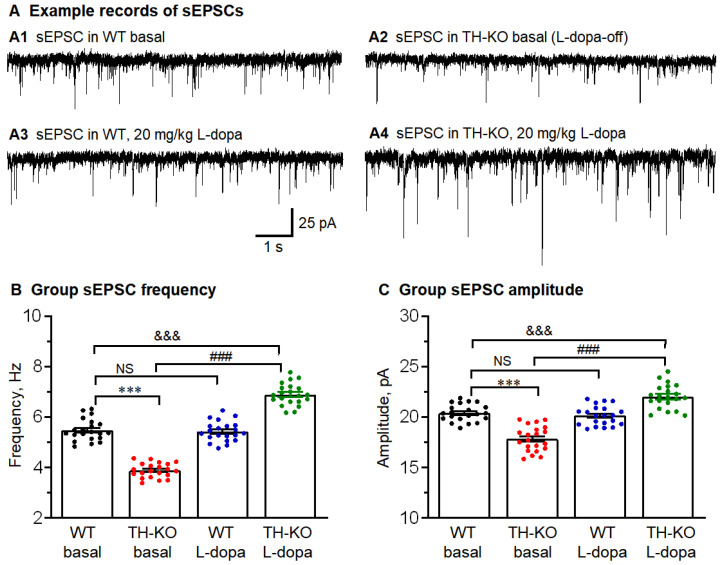
M1 layer 2/3 pyramidal neurons had reduced excitatory synaptic inputs (sEPSCs, including action potential-triggered and action potential-independent glutamatergic inputs) that were reversed by in vivo IP L-dopa treatment. (**A**) Representative raw traces showing sEPSCs recorded in M1 pyramidal neuron: A1: WT-basal; A2: TH-KO-basal (L-dopa-off TH-KO); A3: WT-L-dopa; and A4: TH-KO-L-dopa. (**B**,**C**) Average sEPSC frequency/amplitude bar charts show the decreased average sEPSC frequency and amplitude in TH-KO mice, which were both increased under L-dopa injection in TH-KO mice. All data showed as mean ± SEM, and ***, *p* < 0.001 (frequency: *p* = 0.0002; amplitude: *p* = 0.0003): WT-basal vs. TH-KO-basal; ###, *p* < 0.001 (frequency: *p* = 0.0001; amplitude: *p* = 0.0003): TH-KO-basal vs. TH-KO-L-dopa; &&&, *p* < 0.001 (frequency: *p* = 0.0004; amplitude: *p* = 0.0006): WT-basal vs. TH-KO-L-dopa; NS, not significant: WT-basal vs. WT-L-dopa; two-way ANOVA.

**Figure 7:**
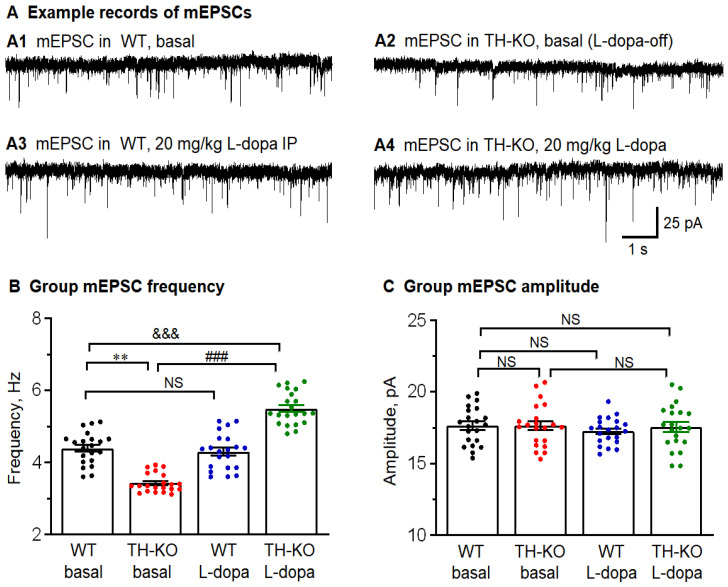
M1 layer 2/3 pyramidal neurons had reduced action potential-independent glutamatergic inputs (mEPSCs) that were reversed by in vivo IP L-dopa treatment. (**A**) Representative raw traces showing mEPSCs recorded in M1 pyramidal neuron: A1: WT-basal; A2: TH-KO-basal; A3: WT-L-dopa; and A4: TH-KO-L-dopa. (**B**,**C**) Average mEPSC frequency and amplitude bar charts show the decreased average sEPSC frequency in TH-KO mice, which was increased under L-dopa injection in TH-KO mice. There is no difference in the mEPSC amplitude between the four groups. All data is shown as mean ± SEM; **, *p* = 0.0013: WT-basal vs. TH-KO-basal; ###, (*p* = 0.0001: TH-KO-basal vs. TH-KO-L-dopa; &&&, *p* = 0.0009: WT-basal vs. TH-KO-L-dopa; NS, not significant: WT-basal vs. WT-L-dopa; two-way ANOVA.

**sEPSCs**: sEPSCs are a mix of action potential-dependent and -independent spontaneous glutamatergic synaptic events and an indicator of pyramidal neuronal and local circuitry activities. Thus, we recorded sEPSCs in M1 pyramidal neurons in WT mice, L-dopa-off TH-KO mice, WT mice treated with 20 mg/kg L-dopa, and TH-KO mice treated with 20 mg/kg L-dopa. As shown in Figure 6 and Figure 7 and Table 3, we made the following observations.

First, for WT mice, sEPSCs had a larger amplitude and a higher frequency than mEPSCs (Figure 6 and Figure 7 and Table 3), which is consistent with the established fact that some sEPSCs in brain slice preparations are action potential-triggered events (these action potentials may be neuronal somata- and axon terminal-originated; also, IP L-dopa treatment did not produce any detectable effect on sEPSCs and mEPSCs (L-dopa also did not induce any detectable behavioral effect in these WT mice, as we have reported before) [17,31].

Second, for L-dopa-off TH-KO mice, sEPSC frequency and amplitude were clearly lower than those for WT mice (Figure 6 and Table 3), indicating fewer action potential-dependent glutamatergic spontaneous synaptic events in TH-KO mice. This is consistent with the lower intrinsic excitability and, hence, there are few spike-dependent spontaneous EPSCs. For IP L-dopa-treated TH-KO mice, sEPSC frequency and amplitude were fully reversed and even surpassed the levels of WT mice (Figure 6 and Table 3).

**mEPSCs:** We recorded mEPSCs in layer 2/3 M1 pyramidal neurons by including 1 μM TTX in the aCSF to block spontaneous action potentials and, hence, action potential-triggered vesicular release. Example records of mEPSCs are shown in Figure 7, and the group values and statistics are listed in Table 3. We made the following observations. First, in WT mice, L-dopa treatment (20 mg/kg, IP) produced no detectable effect on mEPSC amplitude and frequency (Figure 7 and Table 3). Second, in L-dopa-off TH-KO mice, mEPSC frequency was lower than that in WT mice, but mEPSC amplitude was similar to that of WT mice (Figure 7 and Table 3), indicating vesicular glutamate content and postsynaptic receptors were similar in L-dopa-off TH-KO mice and WT mice; the lower frequency of mEPSCs in L-dopa-off TH-KO mice indicates (1) a diminished level of key molecules such as cAMP that facilitate vesicular release from axon terminals, and (2) a lower number of synapses (axon terminals and dendritic spines)—though this is not likely because of the rapid reversal by L-dopa treatment that is probably too fast for anatomical restoration to occur (discussed in Discussion Section). Third, in TH-KO mice, IP L-dopa treatment (20 mg/kg) increased the frequency but not the amplitude of mEPSCs, indicating that IP L-dopa treatment increased, directly and presynaptically, synaptic vesicle release through signaling mechanisms in the presynaptic axon terminals, such as the cAMP-based mechanism, but L-dopa treatment and DA activity do not increase vesicular glutamate content in the axon terminal or postsynaptic glutamate receptors (discussed in the Discussion Section).

These results are qualitatively identical to those of CNO enhancement of mIPSC frequency but not to the amplitude in cAMP-producing Gs-DREADD mice by increasing the vesicular release probability but not changing the content of the synaptic vesicles or postsynaptic GABA receptors [48]. These results are also consistent with the literature stating that intracellular cAMP can increase vesicular neurotransmitter release probability by increasing the vesicle release machinery’s sensitivity to intracellular Ca^2+^ [52,53,54].

### 3.5. Potential Effects of Bath-Applied DA on the Intrinsic Excitability and Synaptic Function of Cortical Pyramidal Neurons in Brain Slices of TH-KO Mice

Data presented in the preceding sections indicated that, in L-dopa-off TH-KO mice, cortical pyramidal neurons have diminished intrinsic excitability and synaptic function, and these deficiencies were reversed by in vivo IP L-dopa treatment. The question now is: How does in vivo L-dopa treatment (20 mg/kg, IP) exert these cellular effects? One possibility is that L-dopa (converted to dopamine after IP injection) acts on cortical pyramidal neurons directly to produce these effects (although literature data on DA’s direct effects on cortical pyramidal neuron excitability are highly conflicting [22,23]) (see also the Introduction and Supplemental Table S1). If so, then DA applied, via bathing solution, to cortical neurons would induce cellular effects on cortical pyramidal neurons as those induced by IP injection of L-dopa. Another possibility is that L-dopa-derived DA primarily acts in the striatum, which then indirectly affects cortical neuron intrinsic excitability and synaptic activity. Empirical data are needed to determine which possibility, or both possibilities, occur in the brain. Our TH-KO mice provide an excellent experimental animal model to test these two possibilities because these mice have hyperfunctional DA receptors that produce larger cellular neurophysiological DA responses that can be more reliably measured and examined [33].

**Intrinsic membrane excitability.** Using the identical current-clamp recording methods described for data in Table 2, we examined the potential effects of bath-applied DA (10 μM) on intrinsic membrane excitability in brain slices from TH-KO mice during the L-dopa-off period, when DA was absent, and DA receptors were hyperfunctional, such that the response to exogenous DA was maximal. As detailed in Table 4, we found that the resting membrane potential (RMP), whole-cell input resistance (R_In_), membrane charging time constant (τ), and spike-firing parameters in M1 cortical layer 2/3 pyramidal neurons were indistinguishable between TH-KO L-dopa-off brain slices perfused with normal aCSF and TH-KO L-dopa-off brain slices perfused with normal aCSF containing 10 μM DA. These results suggest that direct dopaminergic effects on M1 cortical layer 2/3 pyramidal neuron intrinsic excitability are too small to detect even in animals with hyperfunctional DA receptors under our experimental conditions.

**Spontaneous excitatory synaptic transmission.** Following the reasoning described in the preceding paragraphs, here we examined the potential effects of bath-applied DA (10 μM) on sEPSCs in brain slices from TH-KO mice during the L-dopa-off period, when DA was absent, and DA receptors were hyperfunctional, such that the response to exogenous DA was maximal. We found that sEPSC in layer 2/3 pyramidal neurons were indistinguishable between TH-KO L-dopa-off brain slices perfused with normal aCSF and TH-KO L-dopa-off brain slices perfused with normal aCSF containing 10 μM DA. As shown in Table 5, the sEPSC frequency and amplitude were similar in the DA-absent group and the 10 μM DA group. These results suggest that direct dopaminergic effects on cortical glutamatergic synaptic transmission are too small to detect even in animals with hyperfunctional DA receptors.

### 3.6. Cortical Layer 2/3 Pyramidal Neuron Morphology in TH-KO Mice

Finally, we set out to use Golgi staining to examine the morphology of motor cortical layer 2/3 pyramidal neurons in TH-KO mice. But funding ran out before we were able to obtain good staining in the M1 area. However, we obtained good staining in the anterior cingulate cortex. Further, the DA innervation is significantly higher in the anterior cingulate cortex than in the motor cortex (still much lower than that in the striatum) [8]; DA receptor expression is similar in the two areas, or slightly higher in the anterior cingulate cortex [16,55]. Thus, if DA depletion affects cortical neuron morphology, this effect would be more severe in the anterior cingulate cortex than in the motor cortex. Therefore, Golgi staining data from the cingulate cortex can provide useful information about the impact of DA depletion on the anatomy of cortical pyramidal neurons, although our neurophysiology data are from the M1 motor cortex.

**Figure 8:**
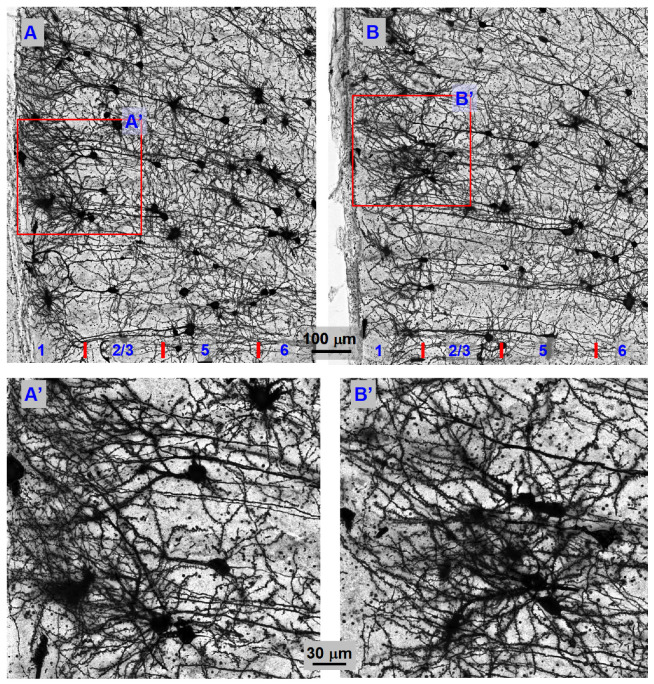
Anterior cingulate cortical pyramidal neuron morphology and the neuronal network are indistinguishable between WT mice and TH-KO mice, indicating normal morphology of cortical pyramidal neurons in TH-KO mice. (**A**,**B**): Compressed display of Golgi-stained anterior cingulate cortical pyramidal neurons from a 7-month-old WT mouse (**A**) and 7-month-old L-dopa-off TH-KO mouse (**B**). These neurons were photographed under a 60× NA1.4 oil objective. (**A’**,**B’**) are larger displays of the boxed areas in (**A**,**B**), showing similarly numerous dendritic spines. Note that no two stained neurons are identical because Golgi staining cannot pre-determine which neuron in the brain to stain (no current method can accomplish this, except in simple non-mammalian animals with a small number of identified neurons), but overall, pyramidal neuron morphology and network pattern are clearly indistinguishable between WT mice and TH-KO mice.

We (three of the authors: MZ, HC, and FMZ) independently examined Golgi-stained mouse ID-blinded sections of the anterior cingulate cortex for five WT mice (7 months of age) and five TH-KO mice (7 months of age), under a 20X objective and then a 60X objective. Working like clinical pathologists, we (MZ, HC, and FMZ) independently evaluated the overall structure, somata, dendrites, and spines to determine if the neurons in the anterior cingulate cortex for five WT mice and five TH-KO mice were different, e.g., do the cingulate cortical pyramidal neurons have shrunk or smaller somata and dendritic trees in one of the two groups of mice? Do cingulate cortical pyramidal neurons in one of the two groups of mice have obviously fewer dendritic spines? However, Golgi staining does not stain the same neurons in different sections, even when these sections are from the same anatomical positions in different brains, making comparison of individual neurons difficult, because different individual neurons in the same layer and area may have differences in dendritic tree size and spine numbers. Additionally, in our Golgi-stained sections, multiple neurons intertwined with each other, making it impractical to quantify the dendritic length and spine number of individual neurons. Thus, we visually and holistically compared the neuronal populations in these tissue sections. Based on our careful examination of these Golgi-stained sections, the three of us independently and unanimously concluded that the neuronal somata, dendrites, and dendritic spine and the neuronal networks in the two sets of cingulate cortical tissue sections (one set from five WT mice, and the other set from five L-dopa-off TH-KO mice) were indistinguishable; two examples are shown in Figure 8A,B.

## 4. Discussion

The main findings of this study are that: (1) whole-brain DA loss leads to reduced cortical pyramidal neuron intrinsic membrane excitability and synaptic function; (2) these impairments are primarily not mediated by loss of DA receptor activity in cortical neurons; (3) direct dopaminergic effects on cortical pyramidal neuron excitability and synaptic function are probably very small; (4) the striatal DA system is the dominant DA system in the brain and indirectly influences/promotes the neuronal activity and function of the cerebral cortex; and (5) DA depletion does not cause cortical pyramidal neuron atrophy.

### 4.1. Whole-Brain DA Loss Leads to Reduced Cortical Pyramidal Neuron Intrinsic Membrane Excitability and Synaptic Function That Are Restored by In Vivo L-Dopa Treatment, but Dopamine Does Not Directly Affect These Neurons

To address our experimental questions, our present study used the global TH KO mouse model [29]. In these mice, DA neurons cannot make L-dopa, leading to a total global lack of DA that renders DA receptors hyperfunctional [30,31]. Hyperfunctional DA receptors amplify behavioral and cellular dopaminergic responses and enable less difficult and more reliable electrophysiological detection of normally modest dopaminergic effects on neuronal excitability [33]. The molecular mechanisms underlying DA receptor hyperfunctionality are not fully established, though higher receptor binding affinity, enhanced receptor-G protein coupling, and enhanced G protein-adenylyl cyclase coupling are probably involved [20,44].

We found that in L-dopa-off TH-KO mice, cortical pyramidal neuron intrinsic membrane excitability, examined in brain slices, was reduced; the synaptic function of these neurons was also reduced. These impairments were reversed by IP L-dopa treatment *before* the mouse was euthanized for brain slice preparation, but bath-applied DA had no detectable effect. These results indicate that the direct dopaminergic effects on cortical pyramidal neurons were too small to detect, and the whole-brain DA loss-induced impairments of cortical pyramidal neuron intrinsic membrane excitability and synaptic function are not due to the lack of DA activity in cortical neurons.

Also, in our study, the intrinsic excitability and synaptic activity of layer 2/3 pyramidal neurons of TH-KO mice after 20 mg/kg L-dopa were higher than those of normal mice. We strongly believe that this is because of the high 20 mg/kg L-dopa dose that also induced motor hyperactivity. We fully expect that lower doses of L-dopa may normalize the intrinsic excitability and synaptic activity of layer 2/3 pyramidal neurons of TH-KO mice.

The lack of detectable effects of directly bath-applied DA on layer 2/3 pyramidal neuron excitability in brain slices in our present study can be understood easily when we take a broad, global, and holistic view of the DA receptor expression in the brain. Literature data and our own data (Figure 3, Table 1) show that the expression level of cortical D1R and D2R is very low, only ~0.07 of the level of striatal D1R and D2R. But even with a highly expressed D1R and D2R, DA’s effects on D1-MSNs and D2-MSNs were difficult to detect, leading to conflicting literature data. We recently established that DA induced only ~1 mV depolarization in D1-MSNs in WT and ~4 mV depolarization in D1-MSNs in TH-KO mice [33]. D1R level in cortical pyramidal neurons is only 0.07 of that of striatal D1-MSNs (Table 1). DA-induced depolarization is likely proportional to the number and functionality of D1Rs. Therefore, DA-induced depolarization is probably ≤0.1 mV in WT cortical pyramidal neurons and ≤0.3 mV in TH-KO cortical pyramidal neurons (Table 6). Slow depolarization with an amplitude of 0.1–0.3 mV is beyond the detection capability of intracellular and patch-clamp recording because the membrane potential may change by more than 0.3 mV in most recorded neurons. Membrane potential changes by ~0.5–1 mV also occur quite often. Only in very high-quality neurons, obtained by highly skilled and very patient electrophysiologists, is the membrane potential stable (change at or <0.5 mV) for a period of ~15 min needed to complete the test. Thus, DA’s direct effects on cortical pyramidal neuron excitability are small and cannot be reliably measured; the functional importance of these direct effects may also be limited. We need to note that fast small events (~down to 0.1 mV) with stereotypical fast rise and slower decay time, such as ionotropic mEPSPs, can be detected by patch-clamp recording: the stereotypical shape of these small events identify themselves as mEPSPs that can be as small as 0.1 mV; although, ~0.5–1 mV is the typical neuronal mEPSP amplitude depending on cell type and the recording conditions that determine cell size, input resistance, and driving force, and hence, mEPSP amplitudes. In contrast, exogenously applied DA-induced small (≤0.5 mV) depolarizations almost certainly have a variable shape such that they cannot be separated from minor recording artifacts.

### 4.2. How Can Striatal Dopaminergic Activity Promote Cortical Pyramidal Neuron Excitability and Synaptic Function?

For the following reasons, we conclude that our observed dopaminergic effects in cortical pyramidal neurons are indirectly triggered by dopaminergic activity in the striatum. First, as described above, in vivo L-dopa treatment reversed the reduction or impairment of cortical pyramidal neuron intrinsic excitability and synaptic function in TH-KO mice, whereas bath-applied DA induced no detectable effect on cortical pyramidal neurons in these mice. In WT mice, neither in vivo L-dopa treatment nor direct bath-applied DA produced any detectable effect on cortical pyramidal neuron excitability and synaptic function. DA receptors are hyperfunctional in TH-KO mice. Thus, a lack of detectable effects of direct bath-applied DA on cortical pyramidal neurons in TH-KO mice strongly indicates that the direct DA effects on cortical pyramidal neurons are too small to detect in normal animals. This is actually consistent with the literature: previous studies reported either no effect, small depolarization, or small hyperpolarization in WT animals (see Supplementary Table S1 for a list of conflicting reports).

Second, both DA innervation and DA receptor expression are low or very low in the cerebral cortex but exceedingly higher in the striatum of rodents and primates, including humans (Figure 4) [8,13,15]. Thus, a lack of detectable direct DA effects on cortical pyramidal neuron excitability and synaptic function is totally expected.

**Figure 9:**
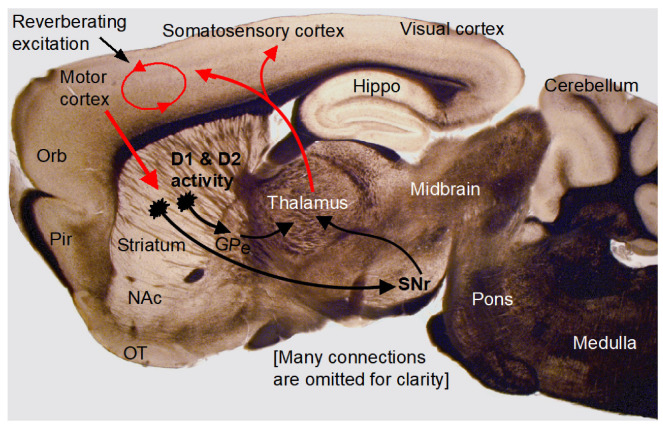
Data interpretation. The photograph shows a mouse brain sagittal section containing the key cortical and subcortical components of the brain, as indicated by the labeling. The superimposed diagram illustrates the cortico–striato–thalamo–cortical positive feedback loop. Arrows indicate key axonal projections (red: excitatory, black: inhibitory). This vast loop is obviously crucial to motor and cognitive brain functions. Because of the dense DA innervation in the striatum and high levels of expression of D1Rs and D2Rs in the striatum, DA can directly affect this loop in the striatum and then indirectly affect the cerebral cortex. More specifically, the strong striatal D1R and D2R activity indirectly increases thalamic neuron activity; the thalamus then increases cortical neuron activity and causes intracortical excitation and reverberating excitation, cortical neuronal excitability, and cortical function. In contrast, due to the very low D1R and D2R expression levels, dopamine induces only very small changes in cortical pyramidal neuron excitability and synaptic activity. Loss of striatal dopaminergic activity causes a loss of the indirect striatal excitation of cortical neurons, leading to a reduction in the intrinsic membrane excitability and synaptic/circuitry function and contributing to the diminished brain function in PD. GPe, globus pallidus external segment; Hippo, hippocampus; NAc, nucleus accumbens; Orb, orbital frontal cortex; OT, olfactory tubercle; Pir, piriform cortex; SNr, substantia nigra pars reticulata.

Third, as illustrated in Figure 9, the strong striatal DA activity can increase thalamic activity that, in turn, can increase cortical neuron activity, activating cortical neuronal networks, increasing synaptic activities including NMDA-R and mGlu1/5, and promoting neuronal activity and spike firing in vivo (Wang & Zhou, manuscript in preparation). In addition to directly enhancing neuronal activity, NMDA-R and mGlu1/5 activity can also activate calcium- and PKC-sensitive adenylyl cyclases [56,57] in cortical neurons and increase intracellular cAMP levels that can further increase neuronal excitability by inhibiting Kir and promote synaptic vesicle release [48], thus promoting cortical neuronal network activity and function (Figure 9). We have documented that Gs-DREADD-induced increase in intracellular cAMP level enhances MSN intrinsic excitability and also increases MSN synaptic vesicular release [48]. Earlier studies by other scientists have also indicated that intracellular cAMP can increase vesicular neurotransmitter release probability by increasing the vesicle release machinery’s sensitivity to intracellular Ca^2+^ [52,53,54].

Fourth, in striatal D1-medium spiny neurons expressing *high* levels of D1-R, we detected a moderate DA-induced increase in intrinsic excitability [33]. Because DA effects are proportional to DA receptor expression levels, it is expected that direct DA effects on cortical pyramidal neurons are very small because of the low levels of D1R expression.

We have published crucial behavioral data on the comparative strength of cortical dopaminergic motor stimulation and striatal dopaminergic motor stimulation: dopaminergic activity in the motor cortex induced no detectable behavioral effect in DA-depleted mice; in striking contrast, dopaminergic activity in the striatum induced robust motor stimulation in these same mice [31].

Fifth, we have performed separate microinjection experiments that show that DA agonist microinjection into the motor cortex induced no behavior or cFos response, whereas DA agonist microinjection into the striatum induced motor activation, cFos activation in striatum and cortex, and cortical neuron spike firing in vivo (Wang & Zhou, manuscript in preparation).

Thus, our new results have a solid anatomical foundation and are consistent with our robust published and unpublished behavioral and neurophysiological data on the DA system [7,17,31,33,48] (Wang & Zhou’s manuscript in preparation).

We need to note that our present study focused on pyramidal neurons. There is a possibility that DA may directly and significantly excite cortical GABAergic interneurons. Indeed, the literature data consistently reported direct DA excitation of cortical GABA interneurons [58,59,60], likely because: (1) GABA interneurons may express relatively higher DA D1/D5 receptors than pyramidal neurons [61]; and (2) GABA interneurons, at least some of them, have less negative RMP and higher input resistance than pyramidal neuron [62,63], such that even a small DA-induced (indirectly) inward current may cause a sufficient depolarization to trigger spike firing, but this DA effect still needs to be viewed in the context the other much stronger synaptic inputs to GABA interneurons.

### 4.3. Cortical Pyramidal Neuron Morphology and Network Appear Normal in DA-Depleted TH-KO Mice

In our Golgi-stained sections of the anterior cingulate cortex, the pyramidal neurons, their dendrites and dendritic spines, and hence, the neuronal network appear normal in TH-KO mice, not distinguishable from WT mice, as examined under a 60× NA1.4 oil objective. We cannot exclude the possibility of ultrastructural abnormalities. However, our observed apparently normal cortical pyramidal neuron morphology is supported by our two additional observations. The first is our extensive behavioral observation: while L-dopa-off TH-KO mice are akinetic, the motor function of these mice was fully restored almost instantaneously upon IP injection of L-dopa (after a delay of ~5 min for IP-injected L-dopa to reach the brain and to be converted to dopamine, i.e., striatal dopamine activity can almost instantaneously restore and stimulate behavior in TH-KO mice). Second, these mice also quickly become cognitively active. It is hard to imagine that these mice can regain their full motor capacity so quickly if the morphology of cortical neurons is impaired, i.e., DA loss-induced akinesia is associated with a loss of normal function of cortical pyramidal neurons rather than structural impairments. This point is further supported by the fact that cortical pyramidal neurons receive very low DA innervation and express very low levels of DA receptors. Third, our extensive observation and experience from maintaining a colony of TH-KO and TH-KO-D2-GFP mice taught us that TH-KO mice can have a normal life span and display good motoric response when daily L-dopa IP injection is very carefully performed such that the mouse receives 3 mg/kg L-dopa daily without needle injury, indicating that the mouse and its brain and cortical pyramidal neurons are normal except the lack of TH gene in DA neurons and hence a lack of DA.

Our finding of normal cortical pyramidal morphology is compatible with data from human PD. First, in the majority of PD patients with moderate motor symptoms, and hence substantial but not severe DA neuron degeneration, their cognition (dependent on neocortical anatomical integrity) is normal at the early and mid-stage of PD [3,64], such that with L-dopa treatment, many of these PD patients can continue, for a few years, to do their jobs, including executive jobs that require normal cognitive function. Thus, neocortical neurons are likely structurally and functionally normal in early and mid-stage PD, although there is currently no data on cortical pyramidal neuron morphology and dendritic spines from early and mid-stage PD brains. Second, 10–20 years after initial diagnosis and at late and end-stage PD, ~25% of PD patients develop cognitive impairment, and an additional ~25% further decline into dementia [64,65]. This is likely because these PD patients may have comorbid neurodegenerative pathologies (Lewy pathology/Lewy dementia) that can more significantly impair neocortical neurons and cognition [65,66]. But data on cortical pyramidal neuron morphology (dendrites and dendritic spines) of PD postmortem brains are lacking, to our knowledge, although cortical pyramidal neuron synapse loss (=spine loss) may occur in late and end-stage PD patients with dementia. Third, in human PD, potential spine loss (no data so far) of cortical pyramidal neurons is likely not caused by loss of DA innervation and dopaminergic activity, but by other pathomechanisms, such as abnormal aggregation of intracellular proteins, e.g., alpha synuclein [65,66]. In existing DA depletion animal models (such as TH-KO, DA toxins), the only pathology is DA loss, and thus, cortical neurons can remain anatomically unaffected.

Our finding of normal cortical pyramidal morphology is also compatible with the literature data from DA depletion animal models. Several studies examined cortical pyramidal neuron morphology in DA depletion animal models, but photographic data in these studies are limited, and hence, the relevant questions are not settled [28]. For example, intracellular staining studies reported that 6-OHDA-induced DA depletion in rats induced no dendritic spine loss in M1 IT and PT pyramidal neurons, no spine morphology change in PT neurons, and a slight spine head enlargement in IT pyramidal neurons [67,68,69]; a Golgi study reported that 6-OHDA-induced DA depletion in rats induced layer 5 pyramidal neuron spine loss in medial PFC (anterior cingulate cortex) but not in the motor cortex [70]; another Golgi study reported that 6-OHDA-induced DA depletion in rats induced no pyramidal neuron spine loss in M1 on the lesioned side [71]; in contrast, an in vivo imaging study reported DA depletion-induced rapid alterations of M1 pyramidal neuron dendritic spines in mice [72]. Our present study made a unique contribution to the field by showing many whole neurons—information and conclusions based on whole neurons are more reliable than those based on dendritic segments.

Finally, we also need to note that the apparently normal morphology of cortical pyramidal neurons in TH-KO mice observed in our present study is consistent with our previous study showing normal dendritic morphology and spine number of striatal medium spiny neurons in TH-KO mice and 6-OHDA-lesioned mice [32]. When DA depletion does not affect the morphology of striatal medium spiny neurons that express high levels of DA receptors and receive dense DA innervation, DA depletion is unlikely to affect the morphology of cortical pyramidal neurons that express very low levels of DA receptors and receive very sparse DA innervation.

### 4.4. Limitations

In our current study, we restricted our patch-clamp recording to M1 layer 2/3 pyramidal neurons due to severely limited funding. Future research needs to record and examine deep-layer pyramidal neurons in M1 and other cortical areas in TH-KO mice to obtain more complete information on dopamine effects in cortical pyramidal neurons. Cortical GABA interneurons also need to be recorded and examined in TH-KO mice. Also, due to limited funding, our Golgi staining data were from the anterior cingulate cortex only, not from M1, where our neurophysiological data were obtained. This mismatch is a significant limitation, although the data from the cingulate cortex are fully valid and useful. We were also unable to quantify the dendritic spines because in our Golgi-stained brain sections, the dendrites of the stained pyramidal neurons in the cingulate cortex intertwined with each other. Future studies need to examine the dendritic morphology in other cortical areas, including the primary motor cortex (M1), and obtain a sufficient number of isolated pyramidal neurons such that dendritic spines of individual neurons can be quantified.

Another limitation of our present study is that only male mice were included. We made this decision because including female mice would double the costs, which we could not afford. But we believe that this limitation is probably minor for the following reasons. First, based on our extensive observations of TH-KO mice, male and female TH-KO mice are equally akinetic when L-dopa is off, and they respond to L-dopa equally. Second, men and women have similar or identical motor skills and cognition, as demonstrated by male and female gymnasts (the muscle force is different between males and females), by male and female scientists, and by male and female justices on the US Supreme Court. Third, male and female PD patients show similar symptoms and receive the same dopaminergic drug treatments. Thus, although male and female brains have a few small (but important) areas serving sex-specific functions, most areas/structures of the brain are similar or identical and serve the same functions, such as motor, cognition, and sensory functions in both male and female animals [73]. We look forward to independent replication studies that overcome these limitations to either validate or invalidate our data presented here.

### 4.5. Implications of Our Present Findings

If independently replicated, our present findings will have important implications for our understanding of the brain DA systems. First, our data contained in this paper show that the cerebral cortical pyramidal neurons receive sparse DA innervation and express low levels of D1Rs and D2Rs, such that no direct DA effect on pyramidal neuron excitability and synaptic function can be detected even in TH-KO mice with hyperfunctional DA receptors. In contrast, striatal medium spiny neurons receive intense DA innervation and express high levels of D1Rs and D2Rs; loss of the strong striatal dopaminergic activity leads to diminished cortical pyramidal neuron membrane excitability and synaptic function, and these cellular deficits are restored by striatal dopaminergic activity following in vivo L-dopa treatment. These findings have enormous implications: for any dopamine-related brain disorders, the striatum, not the cerebral cortex, is the main site of dopaminergic pathogenesis and dopaminergic treatment. Second, our data indicate that the morphology and dendritic spines of cortical pyramidal neurons are probably normal in the total absence of DA. This implies that cortical pyramidal neuron morphology and dendritic spines are probably not suitable biomarkers for pathology and treatment in DA depletion PD models.

Our results also suggest the following: Because D1Rs and D2Rs activate two opposing signaling mechanisms, these two receptors probably should not be expressed in the same neurons to avoid the same neurotransmitter (dopamine) producing two conflicting signals—this is clearly demonstrated by the segregated D1R and D2R expression in the two segregated striatal projection neuron populations [7,33,36]; because cortical pyramidal neurons do not have segregated circuits like the striatal direct and indirect pathways, cortical pyramidal neurons are intrinsically programmed to express only various low amounts of D1Rs and D2Rs: these low levels of D1Rs and D2Rs have very weak effects on cortical pyramidal neurons so that they do not confuse their host neurons; additionally, D1R expression levels are relatively higher than D2Rs such that D1Rs dominate, also avoiding confusing the host neurons.

### 4.6. Concluding Thoughts

Our data indicate that while the sparse DA innervation and low levels of DA receptors in the cerebral cortical pyramidal neurons may directly influence these cortical neurons and have significant functions, the intense DA innervation and highly expressed DA receptors in the striatum generate the dominant dopaminergic brain functions essential for motor control and cognition, and thus this is the main origin of pathogeneses of hypodopaminergic diseases (e.g., PD) and hyperdopaminergic diseases (e.g., cocaine addiction, schizophrenia, and dopaminergic treatment-induced behavioral side effects in PD patients). Together with our prior studies [32,33], our data also indicate that DA depletion does not affect cortical pyramidal neuron morphology and that DA and DA depletion can profoundly influence our motor function and cognition by affecting ion channel activity and synaptic transmission without altering neuronal structure.

## Figures and Tables

**Table 1:** Very low DA level and D1R and D2R levels in rodent and human motor cortexes *.

	M1 cortex/ striatum ratio, in mice (present study)	Frontal cortex/ striatum ratio, in rodents, humans (several papers)	Neocortex/ striatum ratio, in mice (Allen Brain Atlas)	M1 cortex/ striatum ratio, in humans (Hurd 2001)	Frontal cortex/ striatum ratio, in humans (Hall 1994)
DA level	0.064 ± 0.004 (TH immuno-stain, n = 8)	~0.014 (~1:70; DA, by HPLC)			~0.006 (DA, by HPLC), other similar reports
D1R level	0.071 ± 0.005 (immunostain, n = 5)		~0.08 (mRNA ISH)	~0.09 (mRNA ISH)	~0.16 (Bmax autoradiogram)
D2R level	0.062 ± 0.004 (D2-GFP, n = 9)		~0.13 (mRNA ISH)	~0.01 (mRNA ISH)	~0.1 (Bmax autoradiogram)

* In addition to our own data, key data from the literature are listed here for comparison. Human brain data convincingly show D1R is ~twice the D2R levels in the cerebral cortex. Our data failed to detect this D1R vs. D2R difference in the cerebral cortex, likely because the low D1R immunostaining signal and D2R-GFP signal were interfered with non-specific background signal. The literature’s frontal cortex/striatum ratio in rodents and humans were estimated using the precision HPLC measurement data from references [9,10,11,12,13,14]. Hall 1994 [13]; Hurd 2001 [15], ISH, in situ hybridization.

**Table 2:** Effects of IP-injected L-dopa on the electrophysiological properties of M1 pyramidal neurons in WT and TH-KO mice *.

	WT mice Basal	TH-KO mice Basal	WT mice L-Dopa IP	TH-KO mice L-Dopa IP
**RMP (mV)**	−75.45 ± 0.12	−76.83 ± 0.13 ***	−75.43 ± 0.12	−73.87 ± 0.22 ^###^
**Input resistance (MΩ)**	110.7 ± 2.9	80.8 ± 1.8 ***	110.8 ± 1.9	155.1 ± 2.2 ^###^
**Membrane τ (ms)**	16.72 ± 0.4	12.34 ± 0.4 ***	16.54 ± 0.4	23.39 ± 0.3 ^###^
**Rheobase current (pA)**	180.0 ± 5.5	251.4 ± 9.4 ***	181.4 ± 6.7	117.1 ± 5.0 ^###^
**Spike no. evoked by 180 pA**	7.6 ± 0.4	0.7 ± 0.3 ***	7.6 ± 0.3	11.9 ± 0.5 ^###^

* Values are mean ± SEM. *** *p* < 0.001: WT-basal vs. TH-KO-basal (L-dopa-off TH-KO); ^###^ *p* < 0.001: TH-KO-basal vs. TH-KO-L-dopa, two-way ANOVA. Spike no. (number): 180 pA current injection induced, and n = 21 cells in each group. The rheobase current is approximate because the current step was 30 pA, but this approximate parameter is obviously useful; the current ramp is ideal for measuring rheobase current, but it cannot measure other parameters. So our experimental protocol is a practical compromise.

**Table 3:** Effects of IP-injected L-dopa on sEPSC and mEPSC frequency and amplitude in M1 pyramidal neurons in WT and TH-KO mice.

		WT mice basal	TH-KO mice basal	WT mice L-Dopa IP	TH-KO mice L-Dopa IP
**sEPSC** (−70 mV)	Frequency (Hz)	5.5 ± 0.1	3.9 ± 0.1 ***	5.4 ± 0.1	6.9 ± 0.1 ^###^
	Amplitude (-pA)	20.4 ± 0.2	17.8 ± 0.3 ***	20.1 ± 0.2	22.0 ± 0.3 ^###^
**mEPSC** (−70 mV)	Frequency (Hz)	4.4 ± 0.1	3.2 ± 0.1 **	4.3 ± 0.1	5.5 ± 0.1 ^###^
	Amplitude (-pA)	17.6 ± 0.3	17.3 ± 0.3	17.4 ± 0.2	17.5 ± 0.3

Values are mean ± SEM. **, *p* < 0.01, and ***, *p* < 0.001: WT vs. TH-KO basal; ^###^, *p* < 0.001: TH-KO basal vs. TH-KO-L-dopa. WT basal vs. WT L-dopa: no difference for all parameters. n = 21 cells in each group. Two-way ANOVA was employed to detect differences among groups, followed by Tukey’s post hoc comparisons test.

**Table 4:** Lack of detectable effects of bath-applied dopamine on key neurophysiological properties of M1 pyramidal neurons in WT and TH-KO mice.

	WT basal	TH-KO basal	WT bath- applied DA	TH-KO bath-applied DA
**RMP (mV)**	−75.6 ± 0.2	−76.7 ± 0.2	−75.4 ± 0.2	−76.3 ± 0.2
**Input resistance (MΩ)**	107.8 ± 9.3	80.1 ± 1.8	109.8 ± 2.3	81.2 ± 2.0
**Membrane τ (ms)**	16.2 ± 3.1	12.3 ± 0.4	16.4 ± 3.1	12.5 ± 0.4
**Rheobase current (pA)**	187.6 ± 7.4	261.1 ± 7.0	186.2 ± 6.4	257.3 ± 6.5
**Spike no. evoked by 180 pA**	7.5 ± 0.3	0.4 ± 0.2	7.8 ± 0.3	0.5 ± 0.2

N = 16 layer 2/3 pyramidal neurons for each group; each group included 8 mice. 10 μM DA was bath-applied for all neurons. 20 μM DA was also tested on 5 neurons in each group and induced no effect, and the data were not included here. Not different for all parameters for WT basal vs. WT DA and TH-KO basal vs. TH-KO DA, two-way ANOVA. Spike no.: spike number.

**Table 5:** Lack of detectable effect of bath-applied dopamine on sEPSC and mEPSC frequency and amplitude in M1 pyramidal neurons in WT and TH-KO mice.

		WT basal	TH-KO basal	WT bath- applied DA	TH-KO bath- applied DA
**sEPSC** (−70 mV)	Frequency (Hz)	5.32 ± 0.15	3.74 ± 0.09	5.53 ± 0.14	3.82 ± 0.11
	Amplitude (-pA)	19.25 ± 0.61	18.32 ± 0.3	19.74 ± 0.52	18.89 ± 0.2
**mEPSC** (−70 mV)	Frequency (Hz)	4.12 ± 0.12	3.11 ± 0.09	4.25 ± 0.11	3.37 ± 0.10
	Amplitude (-pA)	17.15 ± 0.35	17.06 ± 0.32	17.37 ± 0.33	17.56 ± 0.34

N = 16 layer 2/3 pyramidal neurons for each group; each group included 8 mice. 10 μM DA was bath-applied for all neurons. 20 μM DA was also tested on 5 neurons in each group and induced no effect, and the data were not included here. Not different for all parameters for WT basal vs. WT DA and TH-KO basal vs. TH-KO DA, two-way ANOVA.

**Table 6:** Estimation of DA-induced depolarization in cortical pyramidal neurons based on DA-induced depolarization in D1-MSNs and D1R expression levels in these two neuron types.

	Py Neuron WT Mice	Py Neuron TH-KO Mice	D1-MSN WT Mice	D1-MSN TH-KO Mice
Depolarization	Estimated: 0.07 mV *	Estimated: 0.28 mV *	1 mV (measured: Wang 2025) [33]	4 mV (measured: Wang 2025) [33]
D1 receptor level	0.07 **	0.07 **	1 **	1 **
D1 receptor hyperfunctionality	No	yes: estimated 4X function	No	yes: estimated 4X function

*, ** Pyramidal neuron D1R level and, hence, depolarization are 0.07 of that of D1-MSNs. D2R-mediated hyperpolarization in cortical pyramidal neurons is even smaller because cortical D2R expression is lower than D1R expression. We assume that membrane potential changes are linearly proportional to D1R and D2R expression levels.

## Data Availability

The data presented in this study are available on request from the corresponding author due to ongoing closely related studies.

## Supplementary Materials

The following supporting information can be downloaded at: https://www.mdpi.com/article/10.3390/brainsci16030285/s1; Supplemental Video data: 2 videos (Video S1: before L-dopa; Video S2: after IP L-dopa to both mice: no effect in WT mouse; clear motor stimulation in TH-KO mouse). Supplemental Table S1: Conflicting literature reports on dopamine effects on cortical pyramidal neurons.
