# Supplementary material for: Reduced Cortical Pyramidal Neuron Membrane Excitability and Synaptic Function in Parkinsonian Mice and Their Restoration by L-Dopa Treatment: Indirect Mediation by Striatal Dopaminergic Activity
Source: Brain Sci. 2026 Mar 3;16(3):285. doi: 10.3390/brainsci16030285 (PMC13024266; doi:10.3390/brainsci16030285)
Supplement: Supplementary file 1 [file brainsci-16-00285-s001.zip › brainsci-4134364-Supplementary Table S1.pdf]

Supplemental Table S1. Reported conflicting DA effects on PFC pyramidal neuron spike firing and excitability

Publication	Animal, brain region, layer	DA (μM)	D1 ligand	D2 ligand	Spike firing	R_{In}	Δ mV ↑ or ↓
Penit-Soria et al. 1987	Rat PFC slice, layers 3,5,6	400			↑	↑	
Law-Tho et al. 1994	Rat, ACA* slice, layer 5	50-100			No effect	No effect	Little effect
Geijo-Barrientos & Pastore 1995	Rat ACA slice layers 2/3-6	0.1-10			↓		↓
Yang, Seamans 1996	Rat PFC slice layers 5-6	2-50	SKF38393 (agonist)		↑	↓	
Gulledge & Jaffe 1998	Rat PFC slice, layer 5	0.01-30			↓	↓	
Gulledge & Jaffe 1998	Rat, PFC slice, layer 5			Quinpirole (agonist)	↓		
Gulledge & Jaffe 1998	Rat PFC slice, layer 5		SKF38393 SKF81297		Little or no effect		
Huda et al. 1999	Cat in vivo, PTN **, other neurons	DA: →	Local infusion		↓		
Zhou & Hablitz 1999	Rat PFC slice layer 2/3	20-40			↓ small	↓ small	↓ small
Henze et al. 2000	Monkey PFC slice layer 3	0.5-50			↑		
Gulledge & Jaffe 2001	Rat PFC slice, layer 5	10			↓	↓	
Awenowicz & Porter 2002	Rat in vivo; PTN	DA: →	Local infusion		↓		
Seong & Carter 2012	Mouse; PFC slice layer 5, D1-RFP		SKF81297 (agonist)		↑	↑	↑
Vitrac et al. 2014	Mouse; in vivo, M1 layer 5-6 Py			Quinpirole: local infusion	↑		
Robinson & Sohal 2017	Mouse ACA slice layer 5			Quinpirole: D2 agonist	↑		
Cousineau et al. 2020	Mouse M1 slice layer 5			Quinpirole	No effect		
Swanson et al. 2021	Mouse M1 slice layers 2/3, 5		SCH23390 (antagonist)		Little effect	↑	
Swanson et al. 2021	Mouse M1 slice layers 2/3, 5			Sulpride (antagonist)	Little or no effect	↑ small	
Swanson et al. 2021	Mouse M1 slice layers 2/3, 5; →	DA lesion			Little or no effect	No effect	
Lancon et al. 2021	Mouse ACA slice layers 2/3-5	10-50	SKF81297 D1 agonist		↓	↓	↑
Plateau et al. 2024	Mouse M1 slice layer 5; D1-GFP		SKF81297 D1 agonist		↑ modest	No effect	No effect

(Δ mV ↑ or ↓: ↑-depolarization, ↓-hyperpolarization; *: ACA-anterior cingulate area/cortex; **: PTN-pyramidal tract neuron; in rodents, anterior M1 and ACA are parts of PFC)

References

Penit-Soria J, Audinat E, Crepel F. Excitation of rat prefrontal cortical neurons by dopamine: an in vitro electrophysiological study. Brain Res. 1987 Nov 10;425(2):263-74. doi: 10.1016/0006-8993(87)90509-9. PMID: 3427429.

2: Gulledge AT, Jaffe DB. Dopamine decreases the excitability of layer V pyramidal cells in the rat prefrontal cortex. J Neurosci. 1998 Nov 1;18(21):9139-51. doi: 10.1523/JNEUROSCI.18-21-09139.1998. PMID: 9787016; PMCID: PMC6793538.

Law-Tho D, Hirsch JC, Crepel F. Dopamine modulation of synaptic transmission in rat prefrontal cortex: an in vitro electrophysiological study. Neurosci Res. 1994 Dec;21(2):151-60. doi: 10.1016/0168-0102(94)90157-0. PMID: 7724066.

Geijo-Barrientos E, Pastore C. The effects of dopamine on the subthreshold electrophysiological responses of rat prefrontal cortex neurons in vitro. Eur J Neurosci. 1995 Mar 1;7(3):358-66. doi: 10.1111/j.1460-9568.1995.tb00331.x. PMID: 7773435.

Yang CR, Seamans JK. Dopamine D1 receptor actions in layers V-VI rat prefrontal cortex neurons in vitro: modulation of dendritic-somatic signal integration. J Neurosci. 1996 Mar 1;16(5):1922-35. doi: 10.1523/JNEUROSCI.16-05-01922.1996. PMID: 8774459; PMCID: PMC6578685.

Huda K, Salunga TL, Chowdhury SA, Kawashima T, Matsunami K. Dopaminergic modulation of transcallosal activity of cat motor cortical neurons. Neurosci Res. 1999 Jan;33(1):33-40. doi: 10.1016/s0168-0102(98)00108-4. PMID: 10096469.

Zhou FM, Hablitz JJ. Dopamine modulation of membrane and synaptic properties of interneurons in rat cerebral cortex. J Neurophysiol. 1999 Mar;81(3):967-76. doi: 10.1152/jn.1999.81.3.967. PMID: 10085325.

Henze DA, González-Burgos GR, Urban NN, Lewis DA, Barrionuevo G. Dopamine increases excitability of pyramidal neurons in primate prefrontal cortex. J Neurophysiol. 2000 Dec;84(6):2799-809. doi: 10.1152/jn.2000.84.6.2799. PMID: 11110810.

Gulledge AT, Jaffe DB. Multiple effects of dopamine on layer V pyramidal cell excitability in rat prefrontal cortex. J Neurophysiol. 2001 Aug;86(2):586-95. doi: 10.1152/jn.2001.86.2.586. PMID: 11495934.

Awenowicz PW, Porter LL. Local application of dopamine inhibits pyramidal tract neuron activity in the rodent motor cortex. J Neurophysiol. 2002 Dec;88(6):3439-51. doi: 10.1152/jn.00078.2002. PMID: 12466459.

Seong HJ, Carter AG. D1 receptor modulation of action potential firing in a subpopulation of layer 5 pyramidal neurons in the prefrontal cortex. J Neurosci. 2012 Aug 1;32(31):10516-21. doi: 10.1523/JNEUROSCI.1367-12.2012. PMID: 22855801; PMCID: PMC3429120.

Vitrac C, Péron S, Frappé I, Fernagut PO, Jaber M, Gaillard A, Benoit-Marand M. Dopamine control of pyramidal neuron activity in the primary motor cortex via D2 receptors. Front Neural Circuits. 2014 Feb 28;8:13. doi: 10.3389/fncir.2014.00013. PMID: 24616667; PMCID: PMC3937764.

Robinson SE, Sohal VS. Dopamine D2 Receptors Modulate Pyramidal Neurons in Mouse Medial Prefrontal Cortex through a Stimulatory G-Protein Pathway. J Neurosci. 2017 Oct 18;37(42):10063-10073. doi: 10.1523/JNEUROSCI.1893-17.2017. Epub 2017 Sep 14. PMID: 28912160; PMCID: PMC5647767.

Cousineau J, Lescouzères L, Taupignon A, Delgado-Zabalza L, Valjent E, Baufreton J, Le Bon-Jégo M. Dopamine D2-Like Receptors Modulate Intrinsic Properties and Synaptic Transmission of Parvalbumin Interneurons in the Mouse Primary Motor Cortex. eNeuro. 2020 May 20;7(3):ENEURO.0081-20.2020. doi: 10.1523/ENEURO.0081-20.2020. PMID: 32321772; PMCID: PMC7240291.

Swanson OK, Semaan R, Maffei A. Reduced Dopamine Signaling Impacts Pyramidal Neuron Excitability in Mouse Motor Cortex. eNeuro. 2021 Oct 18;8(5):ENEURO.0548-19.2021. doi: 10.1523/ENEURO.0548-19.2021. PMID: 34556558; PMCID: PMC8525657.

Lançon K, Qu C, Navratilova E, Porreca F, Séguéla P. Decreased dopaminergic inhibition of pyramidal neurons in anterior cingulate cortex maintains chronic neuropathic pain. Cell Rep. 2021 Nov 30;37(9):109933. doi: 10.1016/j.celrep.2021.109933. PMID: 34852233; PMCID: PMC8728690.

Plateau V, Baufreton J, Le Bon-Jégo M. Age-Dependent Modulation of Layer V Pyramidal Neuron Excitability in the Mouse Primary Motor Cortex by D1 Receptor Agonists and Antagonists. Neuroscience. 2024 Jan 9;536:21-35. doi: 10.1016/j.neuroscience.2023.11.006. Epub 2023 Nov 11. PMID: 37952579.
