# Supplementary material for: Reduced Cortical Pyramidal Neuron Membrane Excitability and Synaptic Function in Parkinsonian Mice and Their Restoration by L-Dopa Treatment: Indirect Mediation by Striatal Dopaminergic Activity
Source: Brain Sci. 2026 Mar 3;16(3):285. doi: 10.3390/brainsci16030285 (PMC13024266; doi:10.3390/brainsci16030285)
Supplement: Supplementary file 1 [file brainsci-16-00285-s001.zip › brainsci-4134364-Supplementary Video data_Description.pdf]

Supplemental video data: 2 videos
Video 1: before L-dopa injection, the TH-KO mouse was akinetic, while the WT mouse was active and displaying typical mouse behaviors.
Video 2: IP L-dopa injection clearly induced motor stimulation in the TH-KO mouse but had no observable effect on the WT mouse.
